# Characterizing Symptoms and Identifying Biomarkers of Long COVID in People With and Without HIV: Protocol for a Remotely Conducted Prospective Observational Cohort Study

**DOI:** 10.2196/47079

**Published:** 2023-05-31

**Authors:** Nuria Gallego Márquez, Armaan Jamal, Rowena Johnston, E India Richter, Pamina M Gorbach, Tracy D Vannorsdall, Leah H Rubin, Cheryl Jennings, Alan L Landay, Michael J Peluso, Annukka A R Antar

**Affiliations:** 1 Department of Medicine Johns Hopkins University School of Medicine Baltimore, MD United States; 2 amfAR, The Foundation for AIDS Research New York, NY United States; 3 Department of Epidemiology Fielding School of Public Health University of California Los Angeles, CA United States; 4 Department of Psychiatry and Behavioral Sciences Johns Hopkins University School of Medicine Baltimore, MD United States; 5 Department of Neurology Johns Hopkins University School of Medicine Baltimore, MD United States; 6 Department of Molecular and Comparative Pathobiology Johns Hopkins University School of Medicine Baltimore, MD United States; 7 Department of Epidemiology Johns Hopkins Bloomberg School of Public Health Baltimore, MD United States; 8 Department of Internal Medicine Rush University Medical College Chicago, IL United States; 9 Division of HIV, Infectious Diseases, and Global Medicine University of California California, CA United States

**Keywords:** HIV, SARS-CoV-2, COVID-19, long COVID, post–acute COVID-19 syndrome, postacute sequelae of SARS-CoV-2 infection, prospective observational cohort study, remote study, remote participation

## Abstract

**Background:**

Living with HIV is a risk factor for severe acute COVID-19, but it is unknown whether it is a risk factor for long COVID.

**Objective:**

This study aims to characterize symptoms, sequelae, and cognition formally and prospectively 12 months following SARS-CoV-2 infection in people living with HIV compared with people without HIV. People with no history of SARS-CoV-2 infection, both with and without HIV, are enrolled as controls. The study also aims to identify blood-based biomarkers or patterns of immune dysregulation associated with long COVID.

**Methods:**

This prospective observational cohort study enrolled participants into 1 of the following 4 study arms: people living with HIV who had SARS-CoV-2 infection for the first time <4 weeks before enrollment (HIV+COVID+ arm), people without HIV who had SARS-CoV-2 infection for the first time within 4 weeks of enrollment (HIV−COVID+ arm), people living with HIV who believed they never had SARS-CoV-2 infection (HIV+COVID− arm), and people without HIV who believed they never had SARS-CoV-2 infection (HIV−COVID− arm). At enrollment, participants in the COVID+ arms recalled their symptoms, mental health status, and quality of life in the month before having SARS-CoV-2 infection via a comprehensive survey administered by telephone or on the web. All participants completed the same comprehensive survey 1, 2, 4, 6, and 12 months after post–acute COVID-19 symptom onset or diagnosis, if asymptomatic, (COVID+ arms) or after enrollment (COVID− arms) on the web or by telephone. In total, 11 cognitive assessments were administered by telephone at 1 and 4 months after symptom onset (COVID+ arms) or after enrollment (COVID− arms). A mobile phlebotomist met the participants at a location of their choice for height and weight measurements, orthostatic vital signs, and a blood draw. Participants in the COVID+ arms donated blood 1 and 4 months after COVID-19, and participants in the COVID− arms donated blood once or none. Blood was then shipped overnight to the receiving study laboratory, processed, and stored.

**Results:**

This project was funded in early 2021, and recruitment began in June 2021. Data analyses will be completed by summer 2023. As of February 2023, a total of 387 participants were enrolled in this study, with 345 participants having completed enrollment or baseline surveys together with at least one other completed study event. The 345 participants includes 76 (22%) HIV+COVID+, 121 (35.1%) HIV−COVID+, 78 (22.6%) HIV+COVID−, and 70 (20.3%) HIV−COVID− participants.

**Conclusions:**

This study will provide longitudinal data to characterize COVID-19 recovery over 12 months in people living with and without HIV. Additionally, this study will determine whether biomarkers or patterns of immune dsyregulation associate with decreased cognitive function or symptoms of long COVID.

**International Registered Report Identifier (IRRID):**

DERR1-10.2196/47079

## Introduction

### Background

SARS-CoV-2, the virus that causes acute COVID-19, rapidly spread throughout the world and caused a global pandemic, leading to >6 million confirmed deaths since late 2019 [1]. In early 2020, people with persistent symptoms and new health complications after known or suspected SARS-CoV-2 infection began gathering in online forums and support groups. Working both independently and together, they sounded an alarm in the media for a new, often debilitating, post–COVID-19 syndrome called long COVID [2]. Many other terms for long COVID have been used, including *post-COVID condition, long-haul COVID, post–acute COVID-19, and postacute sequelae of SARS-CoV-2 infection* [3]. Long COVID and related terms generally describe a syndrome encompassing a wide range of symptoms or health conditions that occur after confirmed or suspected SARS-CoV-2 infection that can wax, wane, or persist and can last from weeks to years [3,4].

People with long COVID with interest and skills in research advanced the research agenda by conducting and publishing the first large surveys characterizing the symptoms and time course of long COVID [5]. Dozens of symptoms and sequelae of long COVID have been described since then in survey-based and electronic medical record studies. Commonly reported long COVID symptoms include fatigue, postexertional malaise, difficulties with concentration or thinking (also referred to as “brain fog”), sleep disorders, headache, dyspnea, chest pain, heart palpitations, joint or muscle pain, body aches, anxiety, full or partial loss of taste or smell, among many others. [5-12].

People living with HIV are at a higher risk of severe acute COVID-19 outcomes compared with people without HIV [13-15]. Among people with HIV, those with lower absolute CD4 T cell counts or unsuppressed HIV viral load are at a higher risk of poor acute COVID-19 outcomes [15-17]. However, it is unknown whether living with HIV is a risk factor for long COVID. In addition, it is unknown whether the symptoms, sequelae, or time course of long COVID may differ in people living with HIV compared with people without HIV. People living with HIV are eager to learn the full picture of the risk for long COVID. Although the exact pathophysiological mechanisms contributing to long COVID are currently unclear, some researchers hypothesize that chronic inflammation or immune dysfunction may contribute [18,19]. Chronic inflammation and immune dysfunction are well described in people living with HIV on effective antiretroviral therapy [20]. Whether preexisting inflammation predisposes people living with HIV to long COVID or distorts its presentation needs further investigation. Moreover, some symptoms in individuals with long COVID, such as cognitive symptoms, may overlap with conditions common in people living with HIV, such as HIV-associated neurocognitive disorders.

To investigate long COVID in people living with HIV, we designed a prospective observational cohort study to characterize symptoms, sequelae, and cognitive function formally and prospectively in the 12 months after first SARS-CoV-2 infection in people living with HIV as compared with people without HIV, and as compared with people with no history of SARS-CoV-2 infection both with and without HIV. Because identifying and enrolling a sizeable population of individuals living with HIV with first SARS-CoV-2 infection within the previous 4 weeks would be challenging at any 1 location, and to ensure a study population representative of the demographic and geographic diversity of people living with HIV in the United States, we designed this study to be entirely remotely conducted to allow for enrollment and study participation from any location within the continental United States. Surveys are completed by telephone or on the web, cognitive assessments are administered by telephone, and blood and vital signs are collected via a mobile phlebotomy company. Mobile phlebotomists contracting for this company can meet participants at any location of their choice, for example, home or office.

### Study Objectives

This study aimed to characterize symptoms, sequelae, and cognition formally and prospectively in the 12 months following first SARS-CoV-2 infection in people living with HIV as compared with people without HIV at 1, 2, 4, 6, and 12 months after acute COVID-19 symptom onset or after diagnosis if asymptomatic. In addition, people living with and without HIV with no history of SARS-CoV-2 infection were enrolled as controls and completed study events on a similar timeline. The study also aims to identify blood-based biomarkers or patterns of immune dysregulation that are associated with symptoms or sequelae of long COVID.

Other objectives of this study are as follows:

1. To determine baseline host factors correlated with symptoms and sequelae of long COVID.
2. To understand the impact of SARS-CoV-2 infection on quality of life, sleep, fatigue, cognition, mental health, respiratory function, and behavior in the 12 months following infection in people living with and without HIV.
3. To determine whether abnormal orthostatic vital signs are associated with long COVID.
4. To characterize the host response to SARS-CoV-2 infection in people living with and without HIV, including innate and acquired immune responses and circulating levels of immune signaling molecules.
5. To develop a specimen biorepository that includes data and specimens from people living with and without HIV and with and without prior SARS-CoV-2 infection.

## Methods

### Ethics Approval and Informed Consent

This study was reviewed and approved by the Johns Hopkins University School of Medicine institutional review board (IRB00278774). Written informed consent in English or Spanish was obtained from all participants using either paper consent forms or DocuSign.

### Study Design Overview

**Figure 1:**
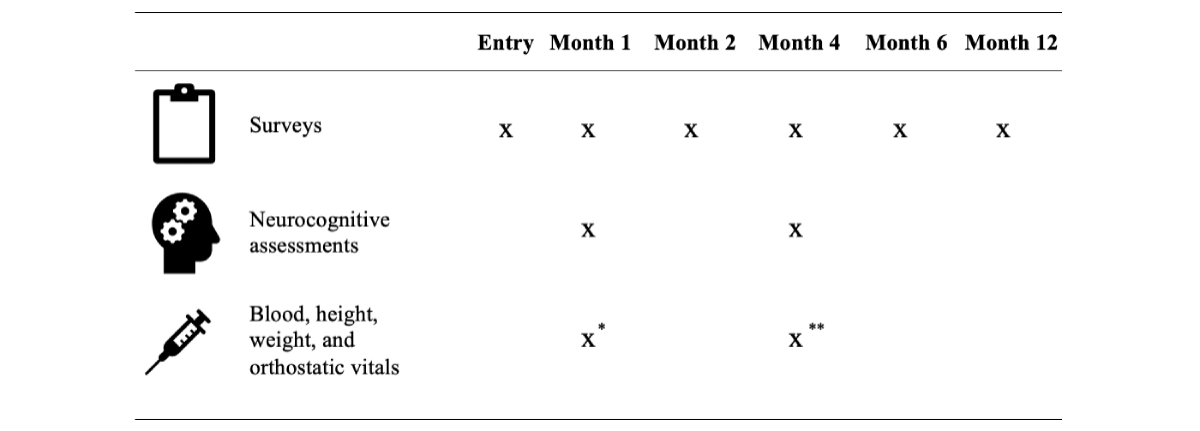
Schedule of study events. *Optional for HIV−COVID− participants; **Only for COVID+ participants.

This study is a prospective, observational cohort study enrolling people living with and without HIV within 4 weeks of first SARS-CoV-2 infection. The study also enrolled people with and without HIV who believed they never had SARS-CoV-2 infection as controls. The study collected health information and specimens from participants at predetermined time intervals through questionnaires, cognitive assessments, and blood draws (Figure 1). This study was designed to enroll participants soon after SARS-CoV-2 infection so that the time course of postacute symptoms and sequelae in the first 12 months could be prospectively investigated and because biomarkers or immune markers of long COVID may only be present early in the postacute period [21]. The study recruited people into the following 4 study arms:

1. HIV+COVID+ arm: people living with HIV with first SARS-CoV-2 infection within the previous 4 weeks.
2. HIV−COVID+ arm: people without HIV with first SARS-CoV-2 infection within the previous 4 weeks.
3. HIV+COVID− arm: people living with HIV who believed they never had SARS-CoV-2 infection.
4. HIV−COVID− arm: people without HIV who believed they never had SARS-CoV-2 infection.

### Study Eligibility

We recruited people with and without HIV, who either had first SARS-CoV-2 infection within the previous 4 weeks or who believed they had never had SARS-CoV-2 infection. Adults aged ≥18 years living in the 48 contiguous United States who had the ability to provide consent, who believed they could complete the study events, and who could communicate by telephone in either English or Spanish were eligible. Further eligibility criteria are presented in Table 1.

**Table 1:** Participant inclusion and exclusion criteria.

Criteria	HIV+COVID+ arm	HIV−COVID+ arm	HIV+COVID− arm	HIV−COVID− arm
Inclusion	Self-reported HIV infection Ability to furnish proof of SARS-CoV-2 infection or reasonable certainty that they had SARS-CoV-2 infection First COVID-19 symptoms or first positive diagnostic COVID-19 test (if asymptomatic) occurred within 28 days before enrollment	Self-reported HIV-negative status^a^ Ability to furnish proof of SARS-CoV-2 infection or reasonable certainty that they had SARS-CoV-2 infection First COVID-19 symptoms or first positive diagnostic COVID-19 test (if asymptomatic) occurred within 28 days before enrollment	Self-reported HIV infection Never tested positive for SARS-CoV-2 and does not believe they have ever had SARS-CoV-2 infection^b^	Self-reported HIV-negative status^a^ Never tested positive for SARS-CoV-2 and does not believe they have ever had SARS-CoV-2 infectionb
Exclusion	A first or only diagnosis of SARS-CoV-2 infection >28 days before contact with study	A first or only diagnosis of SARS-CoV-2 infection >28 days before contact with study	Have tested positive for SARS-CoV-2 or believe that they have had SARS-CoV-2 infection at some point in the past regardless of whether they were tested	Have tested positive for SARS-CoV-2 or believe that they have had SARS-CoV-2 infection at some point in the past regardless of whether they were tested

^a^HIV antigen and antibody test was performed on all participants in this group as part of the study to confirm HIV-seronegative status.

^b^SARS-CoV-2 antinucleocapsid antibody testing was performed as part of this study and must be negative for inclusion in the analyses.

### Recruitment

Participants living with HIV came to know about this study through health care providers, social media, and peers in a number of ways. The study coinvestigators and study team emailed and called HIV clinic directors across the 48 contiguous United States and requested permission to hang study flyers in their clinics. The study principal investigator (PI) and coinvestigators established a network of academic investigators and other people interested in HIV and COVID-19 across the United States and kept them abreast of updated flyers and recruitment updates through regular contact. HIV providers who were colleagues of these people were informed about the study by emails or announcements at provider meetings. Emails were sent to the United States HIV Medicine Association and the Infectious Diseases Society of America’s Exchange listserves (primarily subscribed to by infectious diseases physicians and providers) every 2-4 months with updated flyers and information about the study to increase study referrals. HIV providers who had heard about the study informed their patients with recent SARS-CoV-2 diagnosis about the study and sought permission for the study team to contact them. This study also reached out to established HIV and substance use study cohorts with information on this research study to disseminate to their study cohort participants. Importantly, this study and people living with HIV aiding this study engaged people living with HIV in the community in numerous ways including social networks, email, and social media. The study worked with the Network for Long COVID Justice to amplify dissemination efforts around this study to several social media sites and web-based media sites popular among people living with HIV. The study worked with several community members to disseminate information about this study through social media channels. The study staff asked the participants who were enrolled to ask their friends and contacts who were eligible to contact our study if they were interested; there was no compensation for this recruitment. Advertisements for the study were posted on Facebook and Grindr. Finally, the study investigators received permission to identify people living with HIV with recent positive SARS-CoV-2 tests within the Johns Hopkins Health System via weekly electronic medical record reports. The study staff called these people with an invitation to hear more about the study.

Participants without HIV heard about this study in a number of ways. Advertisements for the study were posted on Facebook and Grindr. The study worked with several community members to disseminate information about this study through social media channels. Participants were recruited through the Johns Hopkins Institute for Clinical and Translational Research recruitment registry, Hopkins Opportunity for Participants Engagement (HOPE) registry. The HOPE registry is a centralized, institutional review board–approved database that contains the name and contact information of people who have taken a SARS-CoV-2 test within the Johns Hopkins Health System and have expressed interest in participating in research studies. The registry is housed in Research Electronic Data Capture (REDCap; Vanderbilt University), and people who have consented to participate in the registry enter their own data into the registry’s web-based intake form, or they have their data collected by recruitment navigators via telephone and entered into the database by HOPE registry navigators. Eligible participants are then matched to each participating study based on the information they have provided and study eligibility criteria. This study’s staff then reached out to the people matched to this study to assess their willingness and eligibility for this study. Physical flyers were posted on the Johns Hopkins medical campus.

A study website was developed to provide information for interested people to self-refer via a web-based REDCap Health Insurance Portability and Accountability Act (HIPAA)–compliant form or by contacting the study directly (via email or phone). This website was advertised on social media platforms such as Facebook (including special interest groups such as for lesbian, gay, bisexual, transgender, queer, and others [LGBTQ+] communities in different cities) and Grindr.

### Consent

A study coordinator based at Johns Hopkins University held a consent conversation with potential participants by telephone or video call, and consent was signed using DocuSign or paper consent forms. All consent activities were conducted in either English or Spanish. The DocuSign process was approved by the institutional review board and is 21 Code of Federal Regulations Part 11–compliant to obtain a secure electronic signature. This process involved sending the consent form to the participant via an email from DocuSign and providing a participant-specific access code that was required when the participant accessed and signed the consent form. Participants were given adequate time to consider the study and ask questions before signing the consent form. When ready to sign, each participant entered their code, verifying that the person signing the consent was the person whom we spoke with previously, and signed the consent within the DocuSign system. Once the participant had electronically signed, the study team member obtaining informed consent would be notified that the electronic form was ready for their signature. Once signing was completed by all parties, both the study team and the participant could download the signed consent as a PDF. The study team also had access to the audit log and the Certificate of Completion. Documentation capturing details of the consent process was maintained on REDCap.

In instances where DocuSign could not be executed or the potential participant declined to use it, the consent conversation happened in person or by telephone or video call and a paper consent was signed. In these cases, participants were provided with a copy of the paper consent document before the consent conversation either via email, fax, mail, or in person. After the consent conversation, the participants were given adequate time to consider the study and ask questions. The participant would sign, date and time the paper informed consent document. The document was then handed, mailed, emailed, or faxed to the consent designee for their signature, and a copy of the double-signed consent document was handed, mailed, emailed, or faxed back to the participant.

### Surveys

Multimedia Appendix 1 [22-48] and Multimedia Appendix 2 provide detailed information about the surveys and the timing of study events. At enrollment, participant demographics, social history, substance use, medical history, medication use, SARS-CoV-2 vaccination status, and HIV history (for people living with HIV only) were recorded. Participants in the COVID+ arms also completed a comprehensive survey on symptoms, mental health, and quality of life via telephone or on the web in the month before having SARS-CoV-2 infection. At each time point after COVID-19 diagnosis (for the COVID+ arms) or after enrollment (for the COVID− arms), participants completed a comprehensive symptom, mental health, and quality of life survey (Multimedia Appendix 1). This survey consisted of several validated tools, which are detailed in the following paragraphs.

Participants indicated the presence and severity (on a scale of “Not at all,” “A little bit,” “Somewhat,” “Very much,” or “Quite a bit”) of 49 long COVID symptoms. Overall, 34 symptoms derive from the FLU-PRO Plus, and 15 additional symptoms were added based on their frequency of report in a Long COVID Patient Led Research Collaborative survey with input from the patient-researcher authors [5,22]. Dyspnea was assessed using the Modified Medical Research Council (mMRC) Dyspnea Scale, which is used to assess the degree of baseline functional disability because of dyspnea [23]. The Breathless, Cough, and Sputum Scale (BCSS) was used to evaluate the respiratory symptoms common in COVID-19 [24]. The survey also included the Insomnia Severity Index (ISI), which is a 7-item questionnaire assessing the participant’s perceived insomnia symptoms and resulting disruption to their lives [25,26]. Participants self-reported the average daily sleep duration in the previous week. Participants completed the Fatigue Severity Scale (FSS), a 9-item questionnaire assessing their perception of the impact of fatigue on their quality of life and activities of daily living [27]. The FSS global fatigue question was used to measure the participants’ global fatigue. At each study event, the participants were asked whether they had received a COVID-19 vaccine or had a new infection with SARS-CoV-2 since the previous study event.

The survey included the Patient Health Questionnaire Depression 8-item (PHQ-8) and Generalized Anxiety Disorder-7 (GAD-7) tool to assess the severity of depressive and anxiety symptoms, respectively [28-30]. Participants also completed the computerized adaptive testing for mental health (CAT-MH), which draws upon a bank of >1000 test items aligned with the Diagnostic and Statistical Manual of Mental Disorders (DSM)-5 criteria.[31-33]. The modules completed included the computerized adaptive diagnostic test for major depressive disorder (CAT-MDD; screener for major depressive disorder), the computerized adaptive diagnostic test–Depression Inventory (CAT-DI; assesses depression severity), and computerized adaptive diagnostic test–Anxiety Inventory (CAT-ANX; assesses anxiety severity). The survey also assessed self-reported cognitive problems using the self-assessment section of the General Practitioner Assessment of Cognition (GPCOG). This tool consists of 6 yes or no items relating to participants’ perceived difficulty with certain activities owing to cognitive limitations compared with a prior time point [34]. We asked participants to evaluate their current ability to complete the tasks compared with their ability before they had COVID-19 or before enrollment.

Quality of life was assessed using the Short Form Survey (SF-36) and the EuroQuol EQ-5D-5L. The SF-36 contains 36 Likert and binary items relating to the participant’s activities and perceptions of health status [35,36]. The EQ-5D-5L assesses general quality of life through 5 dimensions that include mobility, self-care, usual activities, pain and discomfort, and anxiety and depression [37]. The EQ-5D-5L includes a general health question that asks the participant to provide 1 number from 0 to 100 on a visual analog scale representing their overall health [37]. The Brief Resilience Scale was used to assess the perceived ability to bounce back or recover from stress [38]. Stressor-related questions were also asked to assess general stress levels from daily life during the COVID-19 pandemic (Multimedia Appendix 1).

Participants in the COVID+ arms were administered an additional acute COVID-19 survey at 1 or more months after COVID-19 diagnosis in which the participant was asked to recall the presence and severity of the same 49 COVID-19 symptoms described earlier within the first 14 days of symptom onset or positive testing (if asymptomatic). The survey also queried the type of COVID-19 test taken, sample source, COVID-19 exposure, treatment for acute COVID-19, and whether hospitalization or oxygen was required during acute COVID-19. Reinfection was not uncommon in this cohort, and each participant was asked about reinfection (COVID+ arm) or new SARS-CoV-2 infection (COVID− arm). Each individual with a new SARS-CoV-2 infection since enrollment was administered the same acute COVID-19 survey described earlier one or more months after COVID-19 diagnosis to collect information on the acute course of COVID-19. Further information about the surveys used in the study can be found in Multimedia Appendix 1.

### Neurocognitive Assessments

Participants in all arms underwent a telephone-based neurocognitive assessment at 1 and 4 months after acute SARS-CoV-2 symptom onset or diagnosis (COVID+ arm) or after enrollment (COVID− arm). Telephone assessment has been shown to be feasible and provides a valid assessment of cognitive function relative to in-person neurocognitive testing [49-51]. This telephone assessment has been successfully implemented in diverse patient populations, including post–COVID-19 populations [52-54].

A total of 11 cognitive assessments were administered in this study. The Hopkins Verbal Learning Test–Revised (HVLT-R) is a test of new auditory-verbal learning (VL), verbal memory (VM), and recognition discrimination memory (RM) [39,40]. The Oral Trail Making Test part A (OTMT-A) and Oral Trail Making Test part B (OTMT-B) are brief motor-free tests of mental processing speed (part A) and executive functioning requiring sequential set shifting (part B) [39-41]. The Wechsler Adult Intelligence Scale–Fourth Edition (WAIS-IV) Digit Span Forward (DSF) and Digit Span Backwards (DSB) assessments test auditory attention (forward) and working memory (backward) [42-44]. The Calibrated Ideational Fluency Assessment (CIFA) assesses letter-cued and category-cued verbal fluency (VF). Category-cued VF assesses rapid access to semantic information, and letter-cued VF assesses speeded word retrieval in response to phonetic cues [45-47]. The WAIS-IV Information (WAIS-IV IN) test assesses the general fund of knowledge and was only administered once in this study [42-44]. Performance on the WAIS-IV IN is relatively resistant to neurological damage, and scores correlate highly with overall IQ, thereby allowing scores to serve as a proxy for lifelong intellectual functioning [48].

### Mobile Phlebotomist Visits and Clinical Laboratory Testing

Participants in the COVID+ arms had mobile phlebotomy visits at a location of their choice at 1 and 4 months after post–acute COVID-19 symptom onset (or after diagnosis if asymptomatic). Participants in the HIV+COVID− arm had 1 mobile phlebotomy visit at either month 1 or 4, provided that they had not had SARS-CoV-2 infection before the visit. Participants in the HIV−COVID− arm could optionally undergo specimen collection at either month 1 or month 4, provided that they had not had SARS-CoV-2 infection before the visit. Anti–SARS-CoV-2 nucleocapsid immunoglobulin G was measured in all participant specimens, and data from participants in the COVID− arm were excluded from analyses if their specimens tested positive for antinucleocapsid antibodies and they were unaware of SARS-CoV-2 infection.

A mobile phlebotomy company that operates in every state of the United States was engaged to (1) measure participant height and weight, (2) measure orthostatic vital signs, and (3) draw ≤50 mL blood. A blood draw kit was shipped to the participants before the date of the blood draw. The kit included a vital sign sheet, a sodium citrate tube, serum separator tubes, EDTA tubes, and a PAXgene blood RNA tube. All tubes were plastic and labeled with the participant identifier and a space for the date and time of collection. In addition, absorbent sleeves to separate tubes, approved shipping materials including an outer cardboard box, and an inner 95-kPa specimen transport bag with appropriate biohazard labeling were included in the blood draw kit.

For the measurement of orthostatic vital signs, the participants were asked to lie down for 5 minutes before the first blood pressure and heart rate measurement. Participants were then asked to stand up, and after 1 and 3 minutes, additional heart rate and blood pressure measurements were recorded. After the measurement of orthostatic vital signs, blood was drawn from the participant by the phlebotomist. After filling, all tubes were inverted gently 6-8 times. The serum separator tubes were kept upright for 30-60 minutes before centrifugation for 15 minutes at 1100-1300 g by the mobile phlebotomist. The tubes were placed in absorbent sleeves and were shipped overnight using International Air Transport Association–approved procedures to the study laboratory (Rush Clinical Retrovirology Research Laboratory, Chicago). When specimens arrived at the laboratory, designated specimens were sent to the study Clinical Laboratory Improvement Amendments of 1988 (CLIA)–certified testing laboratory (Rush Medical Laboratories, Chicago) for testing. If unforeseen shipping delays occurred and blood was received >48 hours after collection, then some of the clinical testing was not performed owing to stability issues (eg, CD4, complete blood count, lipid panel, and D-dimer). The remaining blood was processed on the same day, and serum, plasma, peripheral blood mononuclear cells (PBMCs), and the PAXgene blood RNA tubes were appropriately frozen and stored. An inventory of the biorepository was created and maintained by the study laboratory using the Laboratory Data Management System (Frontier Science) [55]. The Johns Hopkins REDCap team developed a novel method to directly input biorepository inventory files from the Laboratory Data Management System into the study’s REDCap database for seamless and rapid updating of the biorepository in the central database.

### Biospecimen Testing

The CLIA-certified study testing laboratory ran the following panel of tests: SARS-CoV-2 antinucleocapsid IgG (Abbott Architect), complete blood count with differential, high-sensitivity C-reactive protein, creatine phosphokinase, D-dimer, lipid panel, HIV fourth-generation antigen and antibody testing (for those who self-reported as HIV-seronegative), and CD4 T cell percentage and absolute count (for those who self-reported as living with HIV). Plasma, PBMCs, and serum were also obtained from all samples. If the blood sample was received by the Rush Clinical Retrovirology Research Laboratory >48 hours after collection, the sample was not sent for complete blood count, CD4 panel, lipid panel, or D-dimer according to the clinical laboratory protocol. The blood sample was still processed for plasma and PBMCs, but in cases where the sample did not separate, plasma and PBMCs were not saved. However, serum was obtained from these samples.

Coronavirus antibodies and select hormones and cytokines were quantified in a subset of specimens. The panel of coronavirus antibodies that were quantified included HCoV-229E Spike, HCoV-HKU1 Spike, HCoV-NL63 Spike, HCoV-OC43 Spike, SARS-CoV-1 Spike, SARS-CoV-2 Nucleocapsid, SARS-CoV-2 NTD. SARS-CoV-2 RBD, SARS-CoV-2 Spike, AY.4, B.1.351, BA.2, BA.2+L452M, BA.2+L452R, BA.2.12.1, BA.3, BA.4, BA.5, and CoV-2 Spike. The hormones and cytokines that were tested were based on what might be important in long COVID from the current literature at the time: interferon (IFN)-β, interleukin (IL)-1RA, IL-1α, IFN-λ1, IL-5, IL-8, interferon gamma-induced protein-10 (IP-10), chemokine (C-X-C motif) ligand 9 (CXCL9), macrophage inflammatory protein (MIP)-1β, vascular endothelial growth factor (VEGF), alpha-1-acid glycoprotein 1 (ORM1), MIP-5, T cell immunoglobulin and mucin-domain containing (TIM)-3, Factor D, lipocalin 2 (LCN2), glial fibrillary acidic protein (GFAP), pentraxin 3 (PTX3), Cortisol, IFN-λ2, transforming growth factor (TGF)-β1, ADAMTS13, complement component 5a (C5a), IFN-γ, IL-1β, IFN-λ3, IL-6, C-C motif chemokine ligand 20 (CCL20), C-C motif chemokine ligand 23 (CCL23), and tumor necrosis factor (TNF)-α [21,56-59]. Most of these analytes were measured with validated assays using commercial plates manufactured by Meso Scale Discovery. Results were analyzed using Discovery Workbench (version 4.0.12; Meso Scale Discovery). The antibody kits were provided by the company.

### Data Collection and Management

Database services were provided by the Johns Hopkins University REDCap, a secure, HIPAA–compliant web application for building databases and managing web-based data for research [60]. Almost all data were collected by Johns Hopkins University study staff. A limited number of data points were generated by the mobile phlebotomy company (eg, orthostatic vital signs, height, weight, and status of collection and shipment) and by the study laboratories at Rush University (eg, clinical laboratory test results, status of shipment receipt, and status of sample processing and storage). Study staff at Johns Hopkins University or Rush received data from the mobile phlebotomy company via the phlebotomy secure internet portal and entered it into the REDCap database. The PI of the study was notified of grossly abnormal vital signs or laboratory results and about moderate to high depression or anxiety PHQ-8 and GAD-7 scores. The PI was also notified if the mobile phlebotomist noted any concerning event during the mobile phlebotomy visit. In each case, the PI reached out to the participant to provide basic health advice and guidance and resources for referral to the appropriate health provider.

### Outcomes

#### Primary Outcomes

1. *Time to return to usual health and time to return to usual activities* will be measured using the FLU-PRO Plus instrument where we will assess the days taken to return to usual health and activities after COVID-19 or enrollment.
2. *Fatigue, severity of fatigue, and its effect on activity level* will be measured using the FSS (on a scale of 9-63) and the FSS global fatigue score (on a scale of 0-10) at 1, 2, 4, 6, and 12 months after COVID-19 or enrollment.
3. *Dyspnea* will be measured using the mMRC instrument (on a scale of 0-4) at 1, 4, and 12 months after COVID-19 or enrollment.
4. *Cognitive impairment* will be assessed using age-standardized scores from the 11 cognitive assessments and the GPCOG (on a scale of 0-36) at 1, 4, and 12 months after COVID-19 or enrollment.
5. *Number of symptoms* experienced will be measured using the FLU-PRO Plus instrument and the 11 additional long symptoms in the domains of neuropsychiatry, cardiovascular, and skin (on a scale of 1-5) at 1, 2, 4, 6 and 12 months after COVID-19 or enrollment.

#### Secondary Outcomes

1. *Orthostatic hypotension and orthostatic tachycardia* will be assessed at 1 and 4 months after COVID-19 or enrollment.
2. *Mental health* will be measured via self-reported measures of depressive symptoms and anxiety using the PHQ-8 and GAD-7 assessments at 1, 2, 4, 6, and 12 months after COVID-19 or enrollment and the CAT-MH at 1 and 4 months after COVID-19 or enrollment.
3. *Quality of life* in the domains of physical functioning, physical limitation of role fulfillment, emotional limitation of role fulfillment, energy and fatigue, emotional well-being, social functioning, pain, and general health will be assessed using the SF-36 and the EuroQuol EQ-5D-5L. The *RAND* 36-Item Health Survey 1.0 scoring method will be used to convert these questions into scores of the 8 QoL domains at 1, 4, and 12 months after COVID-19 or enrollment [61,62].
4. *Insomnia* (presence, severity, and patterns of insomnia) and its interference with daily functioning will be assessed by the ISI (on a scale of 0-28) at 1, 2, 4, 6, and 12 months after COVID-19 or enrollment.

### Compensation

All participants were offered compensation for their time and efforts in participating in the study. A compensation of US $25 was offered per blood draw and US $25 per cognitive assessment completed, for a total of up to US $100 (for COVID+ arm) or US $75 (for COVID− arm) if all study events were completed. Participants were compensated in the form of an Amazon or VISA gift card.

### Power Calculation

The target enrollment was as follows: 105 participants in the HIV+COVID+ arm, 105 participants in the HIV−COVID+ arm, 45 participants in the HIV+COVID− arm, and 50 participants in the HIV−COVID− arm. This resulted in a total target sample size of 305 participants overall, 210 of whom would be in the post–COVID-19 arms. Using a 2-tailed α of .05 and 10% estimated proportion of persistent symptoms in the comparator group, we estimated that there was 80% power to detect a difference of 15% in new or worsened symptom prevalence between people living with and without HIV after SARS-CoV-2 infection using a chi-square test of proportions.

### Statistical Analysis

First, we will calculate the prevalence of persistent symptoms across the entire cohort and new or worsening symptoms compared with pre–COVID-19 symptoms in the 2 COVID-19 recovery arms. We will calculate mean and SD as well as median and IQR of the participant symptom score and quality of life by domain, reporting the statistic that is most reflective of the underlying distribution of the data. We will perform a time-to-event analysis to examine the return to baseline health using a Kaplan-Meier curve to graphically demonstrate COVID-19 recovery. To screen for unanticipated clinical syndromes related to COVID-19, we will also perform an exploratory analysis examining the proportion of participants reporting an abnormality in each item on the symptom checklist. In addition, we will perform cross-sectional cluster analyses at each time point to determine how each symptom relates to the others in each study arm at each time point.

After performing the descriptive analyses for each of the comparator arms, we will compare outcomes between the study arms. For binary outcomes such as new or worsened symptoms, we will report an unadjusted prevalence ratio. For symptom score and quality of life domain scores, we will perform unadjusted linear regression. For return to health, we will perform a time-to-event analysis and use a Cox regression model to estimate hazard ratios. In the next stage of the analysis, we will repeat these models but adjust for covariates including potential confounders (age, sex, race, ethnicity, etc) and effect modifiers (vaccination status and severity of illness).

Finally, we will make comparisons between individuals in each study arm with and without persistent symptoms—we will compare various biological analytes outlined in the *Biospecimen Testing* section among people living with HIV with and without persistent symptoms and among people without HIV with and without persistent symptoms and also describe these analytes among people living with and without HIV who have not had SARS-CoV-2 infection.

## Results

The project was funded in early 2021, and recruitment began in June 2021. Data analyses are expected to be completed by summer 2023, and the primary study results are expected to be submitted for publication in 2023. As of February 2023, 387 participants enrolled in this study, 345 of whom completed enrollment and baseline surveys together with at least one other completed study event at month 1, 2, 4, 6, or 12. The 345 participants include 76 (22%) HIV+COVID+, 121 (35.1%) HIV−COVID+, 78 (22.6%) HIV+COVID−, and 70 (20.3%) HIV−COVID− participants.

## Discussion

### Expected Findings

This study will objectively characterize cognition, symptoms, and sequelae in the 12 months after first SARS-CoV-2 infection in people living with and without HIV. We aimed to replicate the demographic and geographic diversity of people living with HIV in the United States by opening enrollment to people who speak English or Spanish living anywhere in the 48 contiguous United States. As the demographics and medical histories of people living with HIV differ in important ways from people without HIV, we will compare post–COVID-19 cognition, symptoms, and sequelae with postenrollment cognition, symptoms, and sequelae in people living with and without HIV who have never had SARS-CoV-2 infection. We anticipate that people living with and without HIV will have broadly overlapping symptoms and sequelae after COVID. However, given that people living with HIV are more likely than people without HIV to have neurocognitive disease, we anticipate that people living with HIV will be more likely to have subjective and objective cognitive difficulties after COVID than people without HIV [63].

The pathophysiology of long COVID is currently not well defined. Proposed mechanisms include perturbations of immune and inflammatory responses during acute COVID-19, persisting reservoirs of SARS-CoV-2 in tissues, reactivation of latent pathogens such as Epstein-Barr virus, alterations of microbiota, autoimmunity and priming of the immune system from molecular mimicry, microvascular blood clotting, endothelial dysfunction, and dysfunctional signaling in the brainstem [18,19,21,56-58,64-72]. This study will be able to provide evidence to support or refute mechanisms that involve immune dysfunction and persistence of SARS-CoV-2 in plasma by testing samples for cytokines and coronavirus antibody and T cell responses. A central biorepository was created with samples from this study, which could be tested in the future for evidence of the reactivation of latent pathogens, complement activation, and autoimmunity.

Research on the relationship between HIV and long COVID is limited. A US-based electronic medical record preprint using data from TriNetX demonstrated that people living with HIV are more likely to report symptoms and sequelae ≥1 months following COVID-19 compared with propensity score–matched HIV-negative individuals [73]. A moderate-sized cohort study in San Francisco found that HIV is a risk factor for neurological long COVID [71]. By contrast, a study in South Africa found that people living with HIV were as likely as HIV-negative individuals to report symptoms 6 months after hospitalization for COVID-19 [74]. Studies like ours that have the ability to link symptoms, sequelae, and objective measures such as cognitive testing to biological analytes are needed to help the field understand why people living with HIV might be more likely to develop long COVID symptoms and sequelae.

### Strengths

This study was designed to meet the needs of people potentially experiencing long COVID who may be juggling fatigue and other symptoms and sequelae with life, work, and family responsibilities. The major strength of this study is its remote and participant-centric design. Participants could complete every study event from any location of their choice, including their home. This also allowed for the recruitment of a diverse participant population from around the United States who spoke either English or Spanish. In addition, the development of a central biorepository for specimen sharing will serve as a resource for investigators interested in the intersection of HIV and long COVID. Another strength of the study was that our surveys were developed in consultation with patient researchers who were experiencing long COVID.

### Limitations

Limitations of the study include study dropout given the extended period of follow-up. To limit dropout, the study staff regularly reminded participants to complete the study events through phone calls, text messages, and emails. Unfortunately, shipping interruptions owing to weather and shipping company errors delayed the delivery of a percentage of specimens and affected which clinical tests could be performed. The survey asking participants to recall symptoms and quality of life in the month before SARS-CoV-2 infection was subject to a recall bias. In addition, as many participants completed the survey on the web in the context of other similar surveys asking about the present time, participants may have reported current rather than pre–COVID-19 symptoms. Questions on alcohol and substance use may have been subject to social desirability bias.

We aim to disseminate results from this study to participants via emails and at least 2 internet calls (with phone numbers and names anonymized by study staff to protect identifying information) so that participants can learn about study findings, ask questions, and provide feedback. We aim to disseminate the results from this study to affected communities by reaching out through the same networks used to advertise the study (social media, community advisory boards, activist groups, informal and formal newsletters, and magazines), ensuring that affected communities hear about science relevant to them.

## Appendix

Multimedia Appendix 1 Additional information about surveys used in the study.

Multimedia Appendix 2 Peer-reviewer report from amfAR, The Foundation for AIDS Research (USA).

## Abbreviations

BCSS: Breathless, Cough, and Sputum Scale
CAT-ANX: Computerized Adaptive Diagnostic Test–Anxiety Inventory
CAT-DI: Computerized Adaptive Diagnostic Test–Depression Inventory
CAT-MDD: Computerized Adaptive Diagnostic Test-Major Depressive Disorder
CAT-MH: Computerized Adaptive Test-Mental Health
CIFA: Calibrated Ideational Fluency Assessment
CLIA: Clinical Laboratory Improvement Amendments of 1988
DSB: Digit Span Backwards
DSF: Digit Span Forward
DSM: Diagnostic and Statistical Manual of Mental Disorders
FSS: Fatigue Severity Scale
GAD-7: Generalized Anxiety Disorder-7
GFAP: glial fibrillary acidic protein
GPCOG: General Practitioner Assessment of Cognition
HIPAA: Health Insurance Portability and Accountability Act
HOPE: Hopkins Opportunity for Participants Engagement
HVLT-R: Hopkins Verbal Learning Test–Revised
IFN: interferon
IL: interleukin
ISI: Insomnia Severity Index
LGBTQ+: lesbian, gay, bisexual, transgender, queer, and others
MIP: macrophage inflammatory protein
mMRC: Modified Medical Research Council
OTMT-A: Oral Trail Making Test part A
OTMT-B: Oral Trail Making Test part B
PBMC: peripheral blood mononuclear cell
PHQ-8: Patient Health Questionnaire Depression 8-item
PI: principal investigator
REDCap: Research Electronic Data Capture
RM: recognition discrimination memory
SF-36: Short Form Survey
TGF: transforming growth factor
TIM: T cell immunoglobulin and mucin-domain containing
TNF: tumor necrosis factor
VF: verbal fluency
VL: verbal learning
VM: verbal memory
WAIS-IV IN: Wechsler Adult Intelligence Scale–Fourth Edition Information test
WAIS-IV: Wechsler Adult Intelligence Scale−Fourth Edition

## Data Availability

The data sets generated or analyzed during this study are not publicly available because of the presence of patient identifying information but are available from the corresponding author upon reasonable request pending institutional review board approval.
