# Supplementary material for: Characterizing Symptoms and Identifying Biomarkers of Long COVID in People With and Without HIV: Protocol for a Remotely Conducted Prospective Observational Cohort Study
Source: JMIR Res Protoc. 2023 May 31;12:e47079. doi: 10.2196/47079 (PMC10234419; doi:10.2196/47079)
Supplement: Multimedia Appendix 1 [file resprot_v12i1e47079_app1.docx]

| **Survey/**  **Instrument** | **Enrollment** | **Month 1** | **Month 2** | **Month 4** | **Month 6** | **Month 12** | **Acute COVID+**  **Course & Treatment^e^** | **Page Numbers/ Citations/** |
| --- | --- | --- | --- | --- | --- | --- | --- | --- |
| Demographic and Social History | X |  |  |  |  |  |  | See page 3 |
| Medical History | X |  |  |  |  |  |  | See page 7 |
| HIV History^a^ | X |  |  |  |  |  |  | See page 11 |
| Changes in Health and Vaccination Survey |  | X | X | X | X | X |  | See page 13 |
| Modified FLU-PRO© + Plus Long COVID Symptoms^b^ | X^c^ | X | X | X | X | X | X**^e^** | See page 16  [22] |
| COVID Hospitalization and Treatment |  |  |  |  |  |  | X**^e^** | See page 20 |
| COVID Re-infection Questions |  | X | X | X | X | X |  | See page 23 |
| Substance Use | X | X |  | X |  | X |  | See page 24 |
| Fatigue Severity Scale | X^c^ | X | X^d^ | X | X^d^ | X^d^ |  | [27] |
| Insomnia Severity Index | X^c^ | X | X^d^ | X | X^d^ | X |  | [25,26] |
| mMRC Dyspnea Scale | X^c^ | X |  | X |  | X |  | [23] |
| GPCOG Reported Cognition |  | X |  | X |  | X |  | [34] |
| Brief Resiliency Scale |  | X |  | X |  | X |  | [38] |
| Breathless, Cough, and Sputum Scale | X^c^ | X |  | X |  | X |  | [24] |
| CAT-MH Depression & Anxiety |  | X |  |  | X |  |  | [31-33] |
| PHQ-8 Depression | X^c^ | X | X | X | X | X |  | [28,29] |
| GAD-7 Anxiety | X^c^ | X | X | X | X | X |  | [30] |
| Neurocognitive Assessment |  | X |  | X |  |  |  | Page 26  [39-48] |
| EQ-5D-5L Quality of life |  | X | X | X | X | X |  | [37] |
| Short Form-36 Quality of life | X^c^ | X |  | X |  | X |  | [35,36] |
| COVID Stressors |  | X |  | X |  | X |  | See page 27 |
| Pain Questionnaire |  | X |  | X |  | X |  | See page 29 |
| Other Sleep and Exercise Related Questions | X | X | X | X | X | X |  | See page 31 |

^a^Only answered by participants living with HIV

^b^Thirty-four symptoms derive from the FLU-PRO© Plus, and fifteen additional symptoms were added based on frequency of report in a Long COVID Patient-Led Research Collaborative survey with input from the patient-researcher authors.

^c^These instruments were only answered by individuals who were COVID+ and asked participants to recall symptoms and quality of life in the month *prior to* COVID diagnosis.

^d^These instruments were only answered by participants that reported feeling week, tired or fatigued (for FSS) or reported not being “very satisfied” with their sleep patterns (for ISI) at that timepoint.

^e^This instrument was administered to participants who were COVID+ at 1 month post infection. It was also administered to anyone in the study that acquired SARS-CoV-2 infection (again or for the first time) after enrollment.

# **Demographic and Social History**

1) What year were you born? __ __ __ __

2) What ethnicity do you consider yourself to be? (mark one)

⃝ Hispanic or LatinX

⃝ Not Hispanic or LatinX

⃝ Don’t Know/Not sure

⃝ Prefer not to answer

3) What race(s) do you consider yourself to be? (Check all that apply)

⃝ American Indian or Alaska Native

⃝ Asian

⃝ Black or African American

⃝ Native Hawaiian or other Pacific Islander

⃝ White

⃝ Other

⃝ Don’t know/Not sure

⃝ Prefer not to answer

4) The next question is about your sex. When I ask about your sex, I am asking about what sex you were determined to be at birth, such as on your original birth certificate. What was your sex assigned at birth?

⃝ Male

⃝ Female

5) The next question asks about gender. Gender is the social part of being male or female. It relates to your self-identity. When I ask about gender, I am asking about whether you regard yourself to be male, female, gender non-conforming, transgender male, transgender female, or if you identify yourself in an additional category. What is your current gender identity? (check one)

⃝ Male

⃝ Female

⃝ Transgender Male

⃝ Transgender Female

⃝ Additional category, specify.

⃝ Prefer not to answer

If “Additional category,” specify _________________

6) What is your sexual orientation? (Mark all that apply)

⃝ Straight/Heterosexual

⃝ Gay/Lesbian/Homosexual

⃝ Bisexual

⃝ Queer

⃝ Two Spirit

7) What is your current weight in pounds: __ __ __ lbs.

8) What is your current height in inches: __ __ in

9) What is the zip code of your primary residence? _________________

10) What is the highest level of education that you completed?

⃝ Never attended school

⃝ Grades 1 through 8

⃝ Grades 9 through 11 (some high school)

⃝ Grade 12 (completed high school or GED)

⃝ Some College, Associates Degree, or Technical Degree

⃝ Bachelor’s Degree

⃝ Any Graduate School or Graduate Degree

⃝ Prefer not to answer

11) What is your marital status?

⃝ Single

⃝ Married or Cohabitating

⃝ Widowed

⃝ Divorced

⃝ Separated

⃝ Prefer not to answer

12) What best describes your current primary residence? [If not ‘house or apartment’ skip to question 14]

⃝ House or apartment

⃝ Nursing home or retirement home

⃝ Hotel or motel

⃝ Dormitory or group home

⃝ Drug recovery or transitional house

⃝ Shelter for people without homes

⃝ Street/outside/tent/encampment

⃝ Abandoned building/squat

⃝ Vehicle (car, van, RV, camper)

⃝ Prefer not to answer

⃝ Other, specify

If “Other,” specify _____________________________

13) How many bedrooms are in the residence? _________________

14) Do you live alone, with a group, family, or multiple families in your primary residence? (If “Alone” skip to question 16).

⃝ Alone

⃝ Group (roommates, transitional housing, recovery residence)

⃝ Single family (parents/guardians, children, grandparents, etc)

⃝ Multiple families (two or more families)

15) How many people are currently living in your household, **including yourself**? A household is defined as all the people that occupy a single housing unit such as a house, apartment, group of rooms, or single room.

16) Are you currently a full time or part time student?

⃝ Yes – Full time

⃝ Yes – Part time

⃝ No

17) What best describes your current employment status?

⃝ Employed – full time

⃝ Employed – part time

⃝ Homemaker or stay at home parent

⃝ Not currently employed

⃝ Retired

⃝ Disabled (not currently employed due to long- or short-term disability)

⃝ Other

If ‘Other,’ specify __________________________

18) Are you an essential worker (Essential workers are exempt from stay at home and shelter in place orders and must report to their place of work. Essential workers include but are not limited to those working in public health/health care, law enforcement, public safety, first responders, food and agriculture, energy and electricity, petroleum, water and wastewater, transportation, public works, communications and IT, and others.)?

⃝ Yes

⃝ No

⃝ Prefer not to answer.

19) Is a member of your household an essential worker? (Essential workers are exempt from stay at home and shelter in place orders and must report to their place of work. Essential workers include but are not limited to those working in public health/health care, law enforcement, public safety, first responders, food and agriculture, energy and electricity, petroleum, water and wastewater transportation, public works, communications and IT, and others.

⃝ Yes

⃝ No

⃝ Prefer not to answer.

20) What was your total household income before taxes in the past 12 months?

⃝ <$25,000.

⃝ $25,001 - $50,000.

⃝ $50,001 - $80,000.

⃝ $80,001 - $130,000.

⃝ >$130,000

⃝ Don’t Know/Not sure.

⃝ Prefer not to answer.

21) Think of a typical day in the month before your COVID-19 symptoms or diagnosis. How many servings of fruit or vegetables did you have in a typical day?

# **Medical History**

1)Do you have HIV?

⃝ Yes

⃝ No

⃝ Not sure

2) Have you ever been diagnosed by a healthcare professional with any of the following medical conditions? Check all that apply

| **Lung** | ◯ No lung disease |
| --- | --- |
| Asthma | ◯ |
| Emphysema or COPD (chronic obstructive pulmonary disease) | ◯ |
| Sarcoidosis | ◯ |
| Pulmonary embolism (blood clot in lungs) | ◯ |
| Other lung disease, specify | ◯ |
| **Heart/cardiovascular** | ◯ No cardiovascular disease |
| Hypertension (high blood pressure) | ◯ |
| Arrhythmia (heart rhythm disorder, SVT, atrial fibrillation, etc) | ◯ |
| High cholesterol or hyperlipidemia | ◯ |
| Coronary artery disease (angina, also known as heart disease) | ◯ |
| Heart attack or myocardial infarction | ◯ |
| Heart failure or cardiomyopathy | ◯ |
| Heart valve disease (aortic stenosis, prior endocarditis, etc) | ◯ |
| Stroke | ◯ |
| POTS (postural orthostatic tachycardia syndrome) | ◯ |
| Other heart or cardiovascular disease, specify | ◯ |
| **Endocrine** | ◯ No endocrine disease |
| Diabetes | ◯ |
| Thyroid, specify | ◯ |
| Other, specify | ◯ |
| **Hematology/blood** | ◯ No hematology disease |
| Deep vein thrombosis (clot in deep vein of arm or leg) | ◯ |
| Sickle cell disease | ◯ |
| Sickle trait | ◯ |
| Other, specify | ◯ |
| **Kidney** | ◯ No kidney disease |
| Chronic kidney disease, if yes, specify if on dialysis or not | ◯ |
| Other, specify | ◯ |
| **Liver and gastrointestinal** | ◯ No liver or GI disease |
| Chronic hepatitis C, if yes, specify if it is cured or not | ◯ |
| Fatty liver disease | ◯ |
| Cirrhosis | ◯ |
| Other gastrointestinal or liver disease, specify | ◯ |
| **Neurologic disease** | ◯ No neurologic disease |
| Other, specify | ◯ |
| **Autoimmune or rheumatologic disease** | ◯ No rheumatologic disease |
| Systemic lupus erythematosus (Lupus) | ◯ |
| Other, specify | ◯ |
| **Brain health** | ◯ No mood disorder |
| Alcohol or substance use disorder | ◯ |
| Anxiety disorder | ◯ |
| Depression | ◯ |
| Post-Traumatic Stress Disorder (PTSD) | ◯ |
| Other, specify | ◯ |
| **Cancer** | ◯No cancer currently or in past |
| Cancer that is in remission or cured, specify | ◯ |
| Cancer that is active or not in remission or currently being treated, specify | ◯ |
| **Immunodeficiency** | ◯ No immunodeficiency |
| Primary immunodeficiency, specify | ◯ |
| Other, specify | ◯ |
| **Other disease or chronic condition not indicated above, please specify** | ◯ |

3) Have you ever had an organ (kidney, liver, lung, etc) or bone marrow transplant?

⃝ Yes, specify

⃝ No

If yes, please specify bone marrow or the organ that you received. _____________________

4) Has anyone in your immediate family (biological parents, siblings and half-siblings, children) ever had a blood clot in a deep vein or lung (deep vein thrombosis (DVT) or pulmonary embolism (PE))?

⃝ Yes

⃝ No

⃝ Not sure

5) Have you received immune suppressive medications within the past 3 months (e.g. for autoimmune or rheumatic disease, therapy for cancer, etc)? Prednisone or other steroids are included here.

⃝ Yes, specify below

⃝ No

List the immune suppressive medications you received in the past 3 months. If prednisone or other steroid, indicate the dosage. ______________________

6) Do you have any current food or drug allergies?

⃝ Yes, specify below

⃝ No

If yes, please list them here ___________________________

7) In the month before your COVID-19 symptoms or diagnosis, were you taking any medications on a regular basis?

⃝ Yes, specify below

⃝ No

Please list the medications you were taking on a regular basis in the month before your COVID-19 symptoms or diagnosis. _____________________________________________

8) Are you currently pregnant?

⃝ Yes

⃝ No

⃝ Don’t know/Not sure

#

# **HIV History**

1) What year were you diagnosed as having HIV infection?

2) What was the lowest CD4 or T cell count that you remember having?

3) Has a doctor or other medical provider ever diagnosed you with any of the following conditions? (select all that apply)

⃝ Pneumocytsis pneumonia (PCP)

⃝ HIV encephalopathy or dementia

⃝ Esophageal candidiasis (yeast infection of throat)

⃝ Cytomegalovirus (CMV)

⃝ Kaposi sarcoma (KS)

⃝ Lymphoma of the brain

⃝ Non-Hodgkin's lymphoma

⃝ Wasting disease (loss of >10% of body weight)

⃝ Toxoplasmosis (“Toxo”)

⃝ Cryptosporidiosis (“Crypto diarrhea”)

⃝ Progressive multifocal leukoenceohalopathy (PML)

⃝ Coccioidomycosis (“Cocci”, valley fever, desert fever)

⃝ Cryptococcosis (“Crypto”, causes meningitis and pneumonia)

⃝ Histoplasmosis

⃝ Mycobacterium avium complex (MAC)

⃝ Recurrent salmonella infections

⃝ Tuberculosis of the lung (TB)

⃝ Tuberculosis outside the lung (TB)

⃝ Thrush (candida, or a yeast infection in the mouth)

⃝ Hairy leukoplakia in the mouth

⃝ Shingles or herpes zoster

⃝ Invasive cervical cancer

⃝ Other, specify

4) Are you currently taking any medications to treat HIV?

⃝ Yes

⃝ No

5) Can you name your current HIV medications? Include any medications you take to prevent other infections if your immune system is weakened from HIV infection (e.g. Bactrim).

6) How many doses of these medications have you missed in the last 30 days?

⃝ 0

⃝ 1-2

⃝ 3-5

⃝ 6 or more

7) What was your most recent viral load? By this we mean the amount of virus in your blood.

⃝ Undetectable (<20 or <50 depending on the test)

⃝ 1 - 200

⃝ 201 - 1000

⃝ >1000

⃝ Not sure

8) Approximately when was your most recent viral load?

⃝ Within the past 3 months

⃝ 3-6 months ago

⃝ 6-12 months ago

⃝ 12-24 months ago

⃝ More than 24 months ago

⃝ Not sure

9) What year was it the last time your HIV viral load was > 200 or not suppressed?

10) What was your most recent CD4 or T cell count?

11) Approximately when was your most recent CD4 or T cell count?

⃝ Within the past 3 months

⃝ 3-6 months ago

⃝ 6-12 months ago

⃝ 12-24 months ago

⃝ More than 24 months ago

⃝ Not sure

11) How frequently do you see the provider who manages your HIV medications?

⃝ Once a year

⃝ Twice a year

⃝ 3-4 times a year

⃝ More than 4 times a year

⃝ Not sure

**Changes in Health and Vaccination Survey**

1) Have you ever received a vaccine to prevent COVID-19?

⃝ Yes

⃝ In a vaccine trial, and I’m not sure if received the vaccine or a placebo shot

⃝ No

⃝ Not sure

2) How many doses of the vaccine did you receive?

⃝ One dose

⃝ Two doses

⃝ Three doses

⃝ Other (specify)

3) Which vaccine did you receive? Complete row for each vaccine endorsed.

|  | **Vaccine Manufacturer** | **Definitely Received** | **Participant in blinded study – do not know if received product** | **When did you receive it?** Prompt for more than one administration for relevant vaccines. |  |  |
| --- | --- | --- | --- | --- | --- | --- |
|  |  |  |  | **Date of First Dose** | **Date of Second Dose** | **Date of Third Dose** |
| ◯ | Pfizer/BioNTech | ◯ | ◯ | __ __ __/__ __/__ __  ◯Day not known | __ __ __/__ __/__ __  ◯Day not known | __ ____/__ __/__ __  ◯Day not known |
| ◯ | Moderna | ◯ | ◯ | __ __ __/__ __/__ __  ◯Day not known | __ __ __/__ __/__ __  ◯Day not known | __ __ __/__ __/__ __  ◯Day not known |
| ◯ | AstraZeneca | ◯ | ◯ | __ __ __/__ __/__ __  ◯Day not known | __ __ __/__ __/__ __  ◯Day not known | __ __ __/__ __/__ __  ◯Day not known |
| ◯ | Johnson & Johnson / Janssen | ◯ | ◯ | __ __ __/__ __/__ __  ◯Day not known | __ __ __/__ __/__ __  ◯Day not known | __ __ __/__ __/__ __  ◯Day not known |
| ◯ | Novavax | ◯ | ◯ | __ __ __/__ __/__ __  ◯Day not known | __ __ __/__ __/__ __  ◯Day not known | __ __ __/__ __/__ __  ◯Day not known |
| ◯ | Do not remember name of manufacturer | ◯ | ◯ | __ __ __/__ __/__ __  ◯Day not known | __ __ __/__ __/__ __  ◯Day not known | __ __ __/__ __/__ __  ◯Day not known |
| ◯ | Other, specify: _______________ | ◯ | ◯ | __ __ __/__ __/__ __  ◯Day not known | __ __ __/__ __/__ __  ◯Day not known | __ __ __/__ __/__ __  ◯Day not known |

4) Have you been to the emergency room, urgent care center, or been hospitalized since your last research clinic visit?

⃝ Yes ⃝ No

4a. If yes, please specify symptoms:

___________

4b. If yes, please specify diagnosis:

___________

4c. If yes, please specify approximate dates of ER or urgent care visit or hospitalization:

___________

5) Have you had any new illness with a fever or significant coughing since your last clinic visit?

⃝ Yes ⃝ No

5a. If yes, please specify symptoms:

___________

5b. If yes, please specify diagnosis:

5b. If yes, please specify date(s) of onset of symptoms:

_________

5c. If yes, please specify length of symptoms:

_________

6) Are you taking any new medications on a regular basis since [autofill last time survey was filled out]?

⃝ Yes ⃝ No

6a. If yes, please list them: __________________________________________________

7) Have you stopped taking any medications on a regular basis since [autofill last time survey was filled out]?

⃝ Yes ⃝ No

7a. If yes, please list them: __________________________________________________

8) Do you have a new medical diagnosis or condition diagnosed since (enrollment, last clinic visit)?

⃝ Yes ⃝ No

8a. If yes, please specify: __________________________________________________

9) Did you develop a blood clot (in your veins, legs, lungs, or elsewhere) since [autofill last time survey was filled out]?

⃝ Yes ⃝ No

9a. If yes, was this clot seen on an imaging study?

⃝ Yes ⃝ No

9b. If yes, approximately what date was this diagnosed?

MM/DD/YYYY: / /

10) Do you have any new food or medication allergies since [autofill last time survey was filled out]?

⃝ Yes ⃝ No

If yes, please list them: __________

11) Have you visited a doctor’s office (in person or virtually) for any new or worsening health complaint since [autofill last time survey was filled out]?

⃝ Yes – if yes, specify reason_______ and type of clinician (primary care, heart specialist, etc) ______

⃝ No

⃝ Unsure

12) Please list the medications you currently take on a regular basis.

13) Are you currently pregnant?

⃝ Yes

⃝ No

⃝ Don’t know/Not sure

⃝ Not applicable

**Modified FLU-PRO© + Plus Long COVID symptoms**

1) In all month 1, 2, 4, 6, and 12 study events, the stem of the question used is: “Have you had any of the following symptoms in the past week?”

At enrollment, the stem of this question used for COVID+ participants is: “Think about the month prior to your COVID-19 symptoms or diagnosis. In a **typical week in the month before your COVID-19 symptoms or diagnosis,** did you have any of the following?”

At enrollment, COVID+ participants are asked to report the presence and severity of symptoms during the first 14 days after symptom onset (or diagnosis if asymptomatic). The stem of this question in this case read: “ Have you had any of the following symptoms **in first 14 days after your COVID-19 symptoms or in the first 14 days of your COVID-19 diagnosis if you never had symptoms**?

| **Symptom** | **Not at all** | **A little bit** | **Somewhat** | **Quite a bit** | **Very much** |
| --- | --- | --- | --- | --- | --- |
| Fever (>100.4 F / >38.0 C or suspected but temperature unknown) | ⃝ Not at all | ⃝ A little bit | ⃝ Somewhat | ⃝ Quite a bit | ⃝ Very much |
| Low grade fever (99.0-100.3 F / 37.2-37.9 C) | ⃝ Not at all | ⃝ A little bit | ⃝ Somewhat | ⃝ Quite a bit | ⃝ Very much |
| Chills or shivering | ⃝ Not at all | ⃝ A little bit | ⃝ Somewhat | ⃝ Quite a bit | ⃝ Very much |
| Felt cold | ⃝ Not at all | ⃝ A little bit | ⃝ Somewhat | ⃝ Quite a bit | ⃝ Very much |
| Felt hot | ⃝ Not at all | ⃝ A little bit | ⃝ Somewhat | ⃝ Quite a bit | ⃝ Very much |
| Sweating | ⃝ Not at all | ⃝ A little bit | ⃝ Somewhat | ⃝ Quite a bit | ⃝ Very much |
| Teary or watery eyes | ⃝ Not at all | ⃝ A little bit | ⃝ Somewhat | ⃝ Quite a bit | ⃝ Very much |
| Sore or painful eyes | ⃝ Not at all | ⃝ A little bit | ⃝ Somewhat | ⃝ Quite a bit | ⃝ Very much |
| Eyes sensitive to light | ⃝ Not at all | ⃝ A little bit | ⃝ Somewhat | ⃝ Quite a bit | ⃝ Very much |
| Ringing in ears | ⃝ Not at all | ⃝ A little bit | ⃝ Somewhat | ⃝ Quite a bit | ⃝ Very much |
| Runny or dripping nose | ⃝ Not at all | ⃝ A little bit | ⃝ Somewhat | ⃝ Quite a bit | ⃝ Very much |
| Congested or stuffy nose | ⃝ Not at all | ⃝ A little bit | ⃝ Somewhat | ⃝ Quite a bit | ⃝ Very much |
| Sinus pressure | ⃝ Not at all | ⃝ A little bit | ⃝ Somewhat | ⃝ Quite a bit | ⃝ Very much |
| Head congestion | ⃝ Not at all | ⃝ A little bit | ⃝ Somewhat | ⃝ Quite a bit | ⃝ Very much |
| Scratchy or itchy throat | ⃝ Not at all | ⃝ A little bit | ⃝ Somewhat | ⃝ Quite a bit | ⃝ Very much |
| Sore or painful throat | ⃝ Not at all | ⃝ A little bit | ⃝ Somewhat | ⃝ Quite a bit | ⃝ Very much |
| Difficulty swallowing | ⃝ Not at all | ⃝ A little bit | ⃝ Somewhat | ⃝ Quite a bit | ⃝ Very much |
| Trouble breathing/shortness of breath | ⃝ Not at all | ⃝ A little bit | ⃝ Somewhat | ⃝ Quite a bit | ⃝ Very much |
| Dry or hacking cough | ⃝ Not at all | ⃝ A little bit | ⃝ Somewhat | ⃝ Quite a bit | ⃝ Very much |
| Wet or loose cough | ⃝ Not at all | ⃝ A little bit | ⃝ Somewhat | ⃝ Quite a bit | ⃝ Very much |
| Chest congestion | ⃝ Not at all | ⃝ A little bit | ⃝ Somewhat | ⃝ Quite a bit | ⃝ Very much |
| Chest pain or pressure, chest tightness or burning in chest | ⃝ Not at all | ⃝ A little bit | ⃝ Somewhat | ⃝ Quite a bit | ⃝ Very much |
| Heart palpitations; funny or fast heartbeat | ⃝ Not at all | ⃝ A little bit | ⃝ Somewhat | ⃝ Quite a bit | ⃝ Very much |
| Constant thirst | ⃝ Not at all | ⃝ A little bit | ⃝ Somewhat | ⃝ Quite a bit | ⃝ Very much |
| Full or partial loss of taste | ⃝ Not at all | ⃝ A little bit | ⃝ Somewhat | ⃝ Quite a bit | ⃝ Very much |
| Full or partial loss of smell | ⃝ Not at all | ⃝ A little bit | ⃝ Somewhat | ⃝ Quite a bit | ⃝ Very much |
| Lack of appetite | ⃝ Not at all | ⃝ A little bit | ⃝ Somewhat | ⃝ Quite a bit | ⃝ Very much |
| Felt nauseous (feeling like you want to throw up) | ⃝ Not at all | ⃝ A little bit | ⃝ Somewhat | ⃝ Quite a bit | ⃝ Very much |
| Vomiting | ⃝ Not at all | ⃝ A little bit | ⃝ Somewhat | ⃝ Quite a bit | ⃝ Very much |
| Stomach ache | ⃝ Not at all | ⃝ A little bit | ⃝ Somewhat | ⃝ Quite a bit | ⃝ Very much |
| Diarrhea | ⃝ Not at all | ⃝ A little bit | ⃝ Somewhat | ⃝ Quite a bit | ⃝ Very much |
| Body aches or pain | ⃝ Not at all | ⃝ A little bit | ⃝ Somewhat | ⃝ Quite a bit | ⃝ Very much |
| Joint pain | ⃝ Not at all | ⃝ A little bit | ⃝ Somewhat | ⃝ Quite a bit | ⃝ Very much |
| Muscle pain | ⃝ Not at all | ⃝ A little bit | ⃝ Somewhat | ⃝ Quite a bit | ⃝ Very much |
| Leg swelling | ⃝ Not at all | ⃝ A little bit | ⃝ Somewhat | ⃝ Quite a bit | ⃝ Very much |
| Tingling, numbness, coldness, or other unusual sensation in skin | ⃝ Not at all | ⃝ A little bit | ⃝ Somewhat | ⃝ Quite a bit | ⃝ Very much |
| Rash | ⃝ Not at all | ⃝ A little bit | ⃝ Somewhat | ⃝ Quite a bit | ⃝ Very much |
| Felt dizzy | ⃝ Not at all | ⃝ A little bit | ⃝ Somewhat | ⃝ Quite a bit | ⃝ Very much |
| Problems with balance | ⃝ Not at all | ⃝ A little bit | ⃝ Somewhat | ⃝ Quite a bit | ⃝ Very much |
| Confusion | ⃝ Not at all | ⃝ A little bit | ⃝ Somewhat | ⃝ Quite a bit | ⃝ Very much |
| Memory problems | ⃝ Not at all | ⃝ A little bit | ⃝ Somewhat | ⃝ Quite a bit | ⃝ Very much |
| Problems with concentration or “brain fog” | ⃝ Not at all | ⃝ A little bit | ⃝ Somewhat | ⃝ Quite a bit | ⃝ Very much |
| Headache | ⃝ Not at all | ⃝ A little bit | ⃝ Somewhat | ⃝ Quite a bit | ⃝ Very much |
| Seizure | ⃝ Not at all | ⃝ A little bit | ⃝ Somewhat | ⃝ Quite a bit | ⃝ Very much |
| Hallucinations or lucid dreaming | ⃝ Not at all | ⃝ A little bit | ⃝ Somewhat | ⃝ Quite a bit | ⃝ Very much |
| Sleeping more than usual | ⃝ Not at all | ⃝ A little bit | ⃝ Somewhat | ⃝ Quite a bit | ⃝ Very much |
| Trouble sleeping or insomnia | ⃝ Not at all | ⃝ A little bit | ⃝ Somewhat | ⃝ Quite a bit | ⃝ Very much |
| Weak or tired or fatigued | ⃝ Not at all | ⃝ A little bit | ⃝ Somewhat | ⃝ Quite a bit | ⃝ Very much |
| Anxiety | ⃝ Not at all | ⃝ A little bit | ⃝ Somewhat | ⃝ Quite a bit | ⃝ Very much |

2) Do you have any other symptom(s) not listed above?

⃝ Yes (specify)

⃝ No

Calculated field: Patient is asymptomatic in the past week.

⃝ Yes

⃝ No

3) Overall, how severe were your symptoms in the past week?

⃝ No symptoms in the past week - skip to 6 if answer was No on last survey; otherwise go to 5 ⃝ Mild

⃝ Moderate

⃝ Severe

⃝ Very Severe

4) Please describe the nature of your symptoms (select all that apply):

☐ Different symptoms appear and disappear at different times

☐ Symptoms tend to appear and disappear at the same time/simultaneously

☐ Symptoms do not fluctuate and are present on a relatively consistent basis

5) Overall, how were your symptoms this week compared to last week?

⃝ Much better

⃝ Somewhat better

⃝ A little better

⃝ About the same

⃝ A little worse

⃝ Somewhat worse

⃝ Much worse

6) How much did your symptoms interfere with your usual activities in the past week?

⃝ Not at all

⃝ A little bit

⃝ Somewhat

⃝ Quite a bit

⃝ Very much

7) Were you able to do/complete your usual (pre-COVID) activities in the past week?

⃝ Yes – if answer was no on last survey, go to 8; otherwise go to 9

⃝ No – skip to 9

8) Approximately what day did you return to your usual (pre-COVID) activities? (If the day cannot be pinpointed, fill in month and year the closest approximate day or ‘99’ if it cannot be guessed.)

MM/DD/YYYY: / /

9) In general, how would you rate your physical health in the past week?

⃝ Poor

⃝ Fair

⃝ Good

⃝ Very Good

⃝ Excellent

10) Were you at your usual (pre-COVID) health status in the past week?

⃝ Yes – if answer was no on last survey, go to 11

⃝ No

11) Approximately what day did you return to your usual health? (If the day cannot be pinpointed, fill in month and year the closest approximate day or ‘99’ if it cannot be guessed.)

MM/DD/YYYY: / /

**COVID Hospitalization and Treatment**

1) To the best of your recollection, what was the sample collection date of the **first time you tested positive for COVID-19**?

2) What type of sample was collected for testing?

⃝ Nose or nasopharyngeal sample

⃝ Throat or mouth swab or oral fluid/saliva

⃝ Blood sample

⃝ Other: Specify

3) Thinking about the first positive test, for what reason were you tested?

⃝ Had symptoms

⃝ No symptoms, but was exposed to someone who had COVID

⃝ No symptoms or exposure, but was being screened for another reason

⃝ Don’t know

⃝ Other, specify:

4) Had you previously had a negative test for COVID-19? Record the collection date of the *most recent* negative test before your positive test.

⃝ Yes, specify:

⃝ No

5) Did you ever have or are you still having symptoms that you think are because of COVID-19?

⃝ Yes, at least some symptoms

⃝ No, did not have any symptoms

6) Did you visit an emergency room or urgent care center or seek advice from a doctor’s office or clinic at any point after the onset of COVID-19 symptoms?

⃝ Yes

⃝ No

7) Were you hospitalized at any point after the onset of COVID-19 symptoms? By this, we mean did you spend 24 hours or more in an emergency room, hospital bed, or observation unit?

⃝ Yes

⃝ No [skip to Q11]

8) Did the healthcare providers ever give you oxygen to help your breathing?

⃝ Yes

⃝ No

⃝ Not sure

9) Were you ever in the intensive care unit or ICU?

⃝ Yes

⃝ No

⃝ Not sure

10) Did you ever require a breathing tube down your throat? In other words, were you on a ventilator?

⃝ Yes

⃝ No

⃝ Not sure

11) In the past 2 months have you been given a new medical diagnosis besides COVID-19?

⃝ Yes (specify)

⃝ No

⃝ Not sure

12) We will provide a list of medications used to treat or prevent COVID-19. **Did you take any of the following for treatment or prevention of COVID-19** within the past 2 months?

For any affirmative response, probe the nature of the participant’s receipt of the agent, including use in the last 3 days. If a participant states that they received the drug as part of a clinical trial, select column B.

| **Medicine or Treatment** | **A.** **Definitely took** | **B.** **May have taken as part of a multi-arm, blinded clinical trial or research study** | **C.** **Definitely did not take** | **D.** **Not sure if taken** |
| --- | --- | --- | --- | --- |
| Monoclonal antibody | ◯ | ◯ | ◯ | ◯ |
| Colchicine | ◯ | ◯ | ◯ | ◯ |
| Convalescent plasma | ◯ | ◯ | ◯ | ◯ |
| Other (indicate which) | ◯ | ◯ | ◯ | ◯ |
| If the patient has been hospitalized since developing COVID-19, continue this table.  If the patient has not been hospitalized, ***SKIP the rest of this table***. |  |  |  |  |
| Remdesivir | ◯ | ◯ | ◯ | ◯ |
| Steroids, such as dexamethasone or prednisone | ◯ | ◯ | ◯ | ◯ |
| Convalescent plasma | ◯ | ◯ | ◯ | ◯ |
| Sarilumab (pronounced  sar-il-ue-mab) | ◯ | ◯ | ◯ | ◯ |
| Tocilizumab (pronounced toe-si-liz-oo-mab) | ◯ | ◯ | ◯ | ◯ |
| Baricitinib (pronounced  bar-i-sye-ti-nib) | ◯ | ◯ | ◯ | ◯ |
| Other, Specify __________ | ◯ | ◯ | ◯ | ◯ |

13) How do you *think* you were exposed to COVID-19?

⃝ Not sure – skip next question

⃝ From a household member

⃝ On the job or at school

⃝ At another public place – store, post office, etc

⃝ At a social or religious gathering

14) Were you wearing a mask during this potential exposure?

⃝ Yes

⃝ No

⃝ Don’t remember

**COVID Reinfection Questions**

1)Have you been diagnosed with COVID-19 or SARS-CoV-2 infection since the last day you completed this survey?

⃝ Yes

⃝ No

2)Have you been diagnosed with re-infection or second/third infection with SARS-CoV-2 or COVID-19 since the last day you completed this survey? This would include getting a positive nasal swab or spit test after recovery from your initial infection, even if you did not have any symptoms. A positive antibody test does not count.

⃝ Yes

⃝ No

3)Approximately what was the date you had your first COVID-19 symptom for this recent past infection?

If you did not experience any symptoms, please indicate the date of your recent positive test.

4)How was this recent COVID-19 infection diagnosed?

⃝ RT-PCR

⃝ Antigen test

⃝ Antibody test

⃝ Clinical diagnosis

⃝ Unsure/Don’t know

⃝ Other (specify)

**Substance Use**

1) In the **past month**, how often did you have a drink containing alcohol?

⃝ Never [skip to Q3]

⃝ Monthly or less

⃝ 2-4 times a month

⃝ 2-3 times a week

⃝ 4 or more times a week

2) In **past month**, how many drinks containing alcohol did you have on a typical day when you were drinking?

⃝ 1 or 2 drinks per day

⃝ 3 or 4 drinks per day

⃝ 5 or 6 drinks per day

⃝ 7 to 9 drinks per day

⃝ 10 or more drinks per day

3) In the **past month**, have you smoked tobacco regularly, meaning cigarettes, cigars, or any product containing tobacco in a hookah?

⃝ Yes

⃝ No [skip to Q5]

4) In the **past month**, on average, how many packs of cigarettes a day did you smoke? (If cigars or hookah only, indicate “none.”

⃝ None

⃝ 1/4

⃝ 1/2

⃝ 1

⃝ More than 1

5) In the **past month**, have you used any form of e-cigarettes containing nicotine (“vaping”)?

⃝ Yes

⃝ No [skip to Q7]

6) In the **past month**, on average, how often have you used any form of e-cigarettes containing nicotine? In other words, how often do you “vape” nicotine products?

⃝ At least once a day

⃝ Nearly every day

⃝ 3 or 4 times a week

⃝ Once or twice a week

⃝ 2 or 3 times total per month

⃝ Once a month

7) In the **past month**, have you used any form of marijuana, THC, or CBD, either by smoking, vaping, edibles, or concentrates?

⃝ Yes

⃝ No

8) In the **past month**, have you used opioids such as heroin, fentanyl, or painkillers?

⃝ Yes

⃝ No

⃝ Don’t know/not sure

⃝ Prefer not to answer

9) In the **past month**, have you used other substances such as cocaine, crystal meth, LSD, or steroids?

⃝ Yes

⃝ No

⃝ Don’t know/not sure

⃝ Prefer not to answer

**Neurocognitive Assessment**

Eleven cognitive assessments were administered in this study. The Hopkins Verbal Learning Test-Revised (HVLT-R) is a test of new auditory-verbal learning (VL), verbal memory (VM), and recognition discrimination memory (RM).[45,46] Oral Trail Making Test parts A (OTMT-A) and B (OTMT-B) are brief, motor-free tests of mental processing speed (part A) and executive functioning requiring sequential set shifting (part B).[45-47] The Wechsler Adult Intelligence Scale – Fourth Edition (WAIS-IV) Digit Span Forward (DSF) and Digit Span Backwards (DSB) assessments test auditory attention (forward) and working memory (backward).[48-50] The Calibrated Ideational Fluency Assessment (CIFA) assessed letter-cued and category-cued verbal fluency (VF). Category-cued verbal fluency assesses rapid access to semantic information, and letter-cued verbal fluency assesses speeded word retrieval in response to phonetic cues.[51-53] The WAIS-IV Information (IN) test assesses general fund of knowledge and was only administered once in this study.[48-50] Performance on the WAIS-IV IN is relatively resistant to neurologic damage, and scores correlate highly with overall IQ, thereby allowing scores to serve as a proxy for lifelong intellectual functioning.[54]The WAIS-IV IN was only administered at the month 1 timepoint.

**COVID Stressors**

This next section will ask you questions about any stress in your life. Your answers are confidential, so please answer to the best of your ability. If you feel uncomfortable answering any question, you can skip it or choose the "prefer not to answer" choice.

At any time IN THE PAST THREE (3) MONTHS:

|  | Yes | No | Don’t know/Unsure | Not applicable |
| --- | --- | --- | --- | --- |
| Have you or someone in your household had to spend more time taking care of children because of school cancellation, loss of daycare, nanny, etc. | ⃝ | ⃝ | ⃝ | ⃝ |
| Have you or someone in your household had to spend more time taking care of elders because you lost caregivers, removed someone from a facility etc.? | ⃝ | ⃝ | ⃝ | ⃝ |
| Has your household lost income from a job or some other source? | ⃝ | ⃝ | ⃝ | ⃝ |
| Have you or anyone in your household lost housing or become homeless? | ⃝ | ⃝ | ⃝ | ⃝ |
| Has your household been unable to pay your rent/mortgage? | ⃝ | ⃝ | ⃝ | ⃝ |
| Have you experienced a change in your health insurance coverage? | ⃝ | ⃝ | ⃝ | ⃝ |
| Has your household had difficulty paying for any basic needs including food, housing, clothing, electricity? | ⃝ | ⃝ | ⃝ | ⃝ |
| Has anyone in your family died that you suspected or knew had COVID-19? | ⃝ | ⃝ | ⃝ | ⃝ |
| Have you earned income? | ⃝ | ⃝ | ⃝ | ⃝ |
| Has your household income changed relative to what your household was earning over a month ago? | ⃝ | ⃝ | ⃝ | ⃝ |
| Did you or any household member have to eat a limited variety of foods due to lack of money? | ⃝ | ⃝ | ⃝ | ⃝ |
| Did you or any household member have to eat fewer meals in a day or smaller meals than usual because there was not enough food? | ⃝ | ⃝ | ⃝ | ⃝ |
| Have you experienced conflict with any household members? (e.g., spouse/partner, roommate, parents, children) | ⃝ | ⃝ | ⃝ | ⃝ |
| Have you had difficulty taking care of your children's needs? (e.g., providing care, supervising schoolwork, and/or balancing their needs with other responsibilities) | ⃝ | ⃝ | ⃝ | ⃝ |
| Have you been able to adequately provide financially for others you support? | ⃝ | ⃝ | ⃝ | ⃝ |
| Have you been able to adequately provide emotionally for others you support? | ⃝ | ⃝ | ⃝ | ⃝ |

**Pain Questionnaire**

1) How much bodily pain have you had during the past week?

⃝ None

⃝ Very mild

⃝ Mild

⃝ Moderate

⃝ Severe

⃝ Very Severe

2) **Have you experienced pain** in the past week that *you did not experience prior to your COVID diagnosis (or enrollment in this study if you never tested positive*)?

⃝ Yes ⃝ No

*If yes…*

Please select where (Select all that apply.)

⃝ Head, face, mouth

⃝ Neck

⃝ Shoulder, arm, hand

⃝ Chest

⃝ Abdomen

⃝ Lower back/spine

⃝ Pelvis

⃝ Anal, perineal, genital

⃝ Buttock, leg, foot

⃝ Other, specify _______

3). What is the character of the pain? (Select all that apply.)

⃝ Throbbing

⃝ Shooting

⃝ Stabbing

⃝ Sharp

⃝ Cramping

⃝ Gnawing

⃝ Hot-burning

⃝ Aching

⃝ Heavy

⃝ Tender

⃝ Splitting

⃝ Tiring-exhausting

⃝ Sickening

⃝ Fearful

⃝ Punishing-cruel

⃝ Other, specify:

4) When you felt pain in the past week, on average what was its intensity?

⃝ 0 – No pain

⃝ 1 – Mild

⃝ 2 – Discomforting

⃝ 3 – Distressing

⃝ 4 – Horrible

⃝ 5 – Excruciating

**Other Sleep and Exercise Related Questions**

1) In the past week, approximately how many **hours of sleep in a 24-hour period** did you typically get? (Answer in increments of 0.5 hours):

2) In the past week, approximately how many hours of moderate or vigorous exercise did you do? (Moderate intensity includes weight lifting, walking, hiking, bicycling, and dancing)

⃝ 0

⃝ 0-1

⃝ 1-2

⃝ 2-3

⃝ >3
