# Supplementary material for: Characterizing Symptoms and Identifying Biomarkers of Long COVID in People With and Without HIV: Protocol for a Remotely Conducted Prospective Observational Cohort Study
Source: JMIR Res Protoc. 2023 May 31;12:e47079. doi: 10.2196/47079 (PMC10234419; doi:10.2196/47079)
Supplement: Multimedia Appendix 2 [file resprot_v12i1e47079_app2.pdf]

amfAR ID: 110180-69-RGCV

Principal Investigator: Annukka Antar, M.D., Ph.D.

Collaborators: Michael Peluso, M.D.; Alan Landay Ph.D.; Steven Deeks, M.D.

Project Title: Symptoms and Biomarkers of Long COVID in People Living with HIV

Reviewer 1

1. **The research question was designed by amfAR (please see RFP excerpt below). Are the applicants answering the question well?**

   Broadly yes, it's a pragmatic study looking at routine clinic cohorts which is the way these rapid studies are being done frankly. The sample size seem small. They do offer a power calculation but as they say, a statistically significant difference in outcome between the groups will depend on the prevalence of each outcome – and they don't define a primary outcome (I'd suggest fatigue).

   It would be better scientifically I think to use a major electronic record-based dataset of thousands of patients in which HIV status was one routinely recorded variable, but such studies are a lot more expensive and time-consuming.

   I'm not an HIV expert.

   As ever in cohort comparisons, the devil will be in how comparable the different groups are in everything except their HIV status. It's hard to tell from the application what measures are being taken to ensure this.

   One aspect of comparability for example is how the acute illness is managed. It MAY be that because HIV is a risk factor, someone with HIV will be more likely to have their symptoms taken seriously and treated promptly eg with oxygen. Some long covid may be due to early prolonged hypoxia.

2. **How useful will the answers be to PWH, or care providers?**

   I think useful (if preliminary due to small sample). If I had HIV and developed long covid symptoms I'd like to know how they'd play out.

3. **Which specific changes should amfAR ask the applicants to make?**

   Justify the comparability of groups at baseline.

RESPONSE:

We thank Reviewer 1 for their insight and reasoned feedback, which we respond to below:

1) Concerns about sample size. We agree that sample size is a limiting factor, and so we have maximized the sample size as much as allowable within the constraints of the available budget. It would have been our preference to include a larger number of patients in the study,

but this is not feasible given the budget constraints. However, we agree with the reviewer that this grant is likely to answer some key questions regarding COVID pathophysiology in people with HIV, and it will also produce valuable preliminary data that the investigators and amfAR could use in ongoing studies of COVID in this special population. That being said, we do think we are adequately powered to answer the questions outlined in this proposal, and that the exploratory outcomes will yield early data to contribute to additional projects, potentially in combination with other cohorts.

2) Lack of a clear primary outcome measure. Reviewer 1 correctly pointed out that we did not clearly specify the primary outcome in the initial proposal. We will evaluate the following outcomes:

- Primary outcomes:
  - Time to return to usual health and time to return to usual activities as measured using the FLU-PRO instrument.
  - Fatigue: presence/absence of fatigue at 1 and 4 months post-symptom onset, severity of fatigue and its effect on activity level as measured using the Fatigue Severity Scale at 1 and 4 months.
  - Dyspnea: Modified Medical Research Council (mMRC) instrument at 1 and 4 months.
  - Cognitive impairment in the domains of learning and memory (Rey Auditory Verbal Learning Test-RAVLT), attention/working memory (digit span task-forward & backward), phonemic and semantic fluency (letter-guided and category-guided fluency tests), and attention/executive function (oral Trail Making Test Part A and B) at 1 and 4 months.
  - Number of symptoms experienced at 1 and 4 months using FLU-PRO and additional COVID-19 specific symptoms in domains of neuropsychiatry, cardiovascular, and skin.
- Secondary outcomes:
  - Dysautonomia: Orthostatic hypotension and orthostatic tachycardia at 1 and 4 months.
  - Mental health: Self-reported measures of depressive symptoms and anxiety at 1 and 4 months using PHQ-9 and GAD-7 and the Computer Adaptive Test-Mental Health (CAT-MH).
  - Quality of life at 1 and 4 months in the domains of vitality, physical functioning, bodily pain, general health perceptions, physical and emotional and social role functioning, as assessed using the Short Form-36
  - Insomnia: Presence, severity, and patterns of insomnia, and its interference with daily functioning at 1 and 4 months, as assessed by the Insomnia Severity Index.
- Exploratory outcomes: biomarkers of inflammation and immune dysfunction as outlined in proposal, new medical diagnoses after COVID-19

In a recently published study of 669 Swiss COVID-19 outpatients, 32% had one or more symptoms at 30-45 days from diagnosis and another ~20% were not able to be contacted at that time interval (Nehme M. et al Ann. Int. Med. 12/8/2020). Fatigue as a primary outcome is a welcome suggestion as it was the most common persistent symptom in the Swiss cohort as well as two other cohorts that measured symptoms 3-4 weeks after diagnosis (Tenforde M. et al.

MMWR. 7/24/2020, Blair P. et al. medRxiv. 9/3/2020). In the Swiss cohort, 12% of participants who were reached reported fatigue at 30-45 days after diagnosis. As we outline in the proposal, we believe that a 15% difference between groups would be clinically significant and we have 80% power to detect a difference of this magnitude using the current sample size. A larger sample would allow us to detect a smaller difference in the proportion between groups, but it is not clear that a difference of lesser magnitude would be clinically important.

3) The importance of ensuring comparability between HIV+ and HIV- COVID survivors. We agree with the reviewer that establishing comparability between HIV+COVID+ and HIV-COVID+ groups in our cohort will be challenging but critical to the success of the project. We have been planning carefully in order to address this concern.

The primary focus of recruitment will be HIV+ COVID+ individuals, because overall there will be fewer of these individuals. At the end of each 2-week block of time after enrollment begins, we will assess the number of HIV+COVID+ participants recruited in that block. In the next 2-week block of time, we will continue to recruit HIV+COVID+ participants while conducting targeted recruitment of HIV-COVID+ participants that roughly match (in numbers recruited, age, sex, and race/ethnicity) the HIV+COVID+ participants recruited in the preceding 2-week block.

This targeted recruitment of HIV-COVID+ participants will occur at the sites that have local IRB approval to screen for COVID-19 positive cases (Johns Hopkins, UCSF, Rush University, and select other sites will be set up to do this at the discretion of the local collaborators). We will also match based on severity of acute COVID-19 (ICU/hospitalized/never hospitalized) within the first 2-3 weeks of diagnosis. This allows flexibility of HIV+COVID+ enrollment since this group will be most difficult to find, and staggers enrollment of a targeted HIV-COVID+ group to be able to roughly match on age, sex, race/ethnicity, and severity of acute COVID-19.

We agree that an electronic health record-based study of thousands of patients is theoretically the best way to examine differences between PWH and HIV-negative people, but this is true only if the outcome is one that is well-captured in the electronic health record such as death or intubation. In our experience, symptoms like fatigue, insomnia, and brain fog are poorly captured by clinicians in real-life practice - despite requirements for review of systems documentation - or specifically recorded in the electronic record. In addition, because of care fragmentation in the U.S., many participants may have a positive test from a health department or urgent care center, follow up once with a physician by telemedicine in the ER after testing positive, and then recover on their own at home with little contact with the medical system in the months following COVID-19 diagnosis. Many patients with long COVID describe difficulty finding physicians who will listen to and treat their symptoms and so access to care – even with insurance – is a limiting factor with this approach. Indeed, many of the study participants in the local studies run by the co-PIs have been unable to engage with the healthcare system despite the presence of these issues.

Reviewer 2

1. **The research question was designed by amfAR (please see RFP excerpt below). Are the applicants answering the question well?**

   The outstanding group of applicants are well poised to deliver on this amfAR research question, and indeed with current rates of infection across the country, the numbers of volunteers should not be hard to recruit, but the study needs to start soon – and everything seems to be in place to start without issue.

The applicants should make more effort to recruit women, minorities, people with co-existing conditions, old and young.

If the applicants can develop a strategy to recruit asymptomatic SARS-CoV-2 infected participants, that would strengthen the cohort.

2. **How useful will the answers be to PWH, or care providers?**

There are already large studies such as from Barcelona asking similar questions, but I think this cohort will be of value to understand COVID-19 in PWH.

3. **Which specific changes should amfAR ask the applicants to make?**

The applicants should make more effort to recruit women, minorities, people with co-existing conditions, old and young people alike.

The applicants should strategize on the possibility of recruitment of asymptomatic SARS-CoV-2 carriers.

The applicants should also be encouraged to develop plans to manage those who receive COVID vaccines, on their studies.

The applicants should have some plan on quality control between sites, sharing of anonymized samples etc.

RESPONSE:

We thank Reviewer 2 for their suggestions to improve the study. Responses to their comments are listed below:

1) Issues regarding selection of the COVID+ groups. As Reviewer 1 also points out, selection of the two COVID+ groups is critical to the success and interpretability of the study. We strongly agree with Reviewer 2 that the population recruited in the proposed study should reflect the diversity of people living with HIV in the United States. Diversity is also critical for a study of long COVID given that differences in severity of acute disease have been noted by sex, age, race/ethnicity, and comorbidities.

As outlined in the response to Reviewer 1, recruitment of HIV-COVID+ participants will be staggered by 2 weeks behind HIV+COVID+ participants in order to roughly match the groups by age, sex, race/ethnicity, and severity (including asymptomatic). HIV+COVID+ recruitment will occur first in this staggered scenario since this is the population that will be most difficult to recruit. Each month in the first 6 months of recruitment we will examine the age, sex, race/ethnicity, and severity stratification in both COVID+ groups to ensure that both groups reflect the sex, age, race/ethnicity diversity of PWH in the US. The HIV+COVID- group is smaller and will be recruited in a similarly staggered way to roughly match the HIV+COVID+ group by age, sex, race/ethnicity, and co-morbidities.

2) Concerns about consistency of study administration between sites. This was a major consideration in our design of the study and one that we thought carefully about when we decided on the proposed study structure. We anticipate that quality control between sites should be easily achieved since surveys and cognitive tests will be administered by the same JHU study coordinator and applied consistently no matter the site of recruitment. Blood draws will be contracted to the same mobile phlebotomy company that has a national footprint. The central coordination of the study will be crucial to ensure that we can make valid comparisons across participants from diverse geographic areas, in addition to ensuring that the study implementation does not become burdensome on local sites who will be engaged in their own COVID research and clinical activities.

3) Need for a robust specimen sharing system. The development of a central biorepository for specimen sharing is one of the major contributions of this amfAR study. We will work with co-investigator Dr. Alan Landay to establish a biorepository of de-identified specimens that is freely available to the HIV and COVID research communities upon request and institutional review board approval. Dr. Landay is PI of the Rush University COVID biorepository and is a Scientific Director of the ACTG Laboratories. All samples will be shipped to, processed by, and stored in his laboratory. We will establish a procedure for requests for biospecimens similar to that at Rush and currently used in the SCOPE and LIINC studies at UCSF, which have supported hundreds of research collaborations over the last two decades and dozens over the last year. This will result in multiple opportunities for further collaboration using the core amfAR repository.

4) Participants with asymptomatic infection. Recruiting asymptomatic participants is important, as estimates suggest than over one-third of individuals with SARS-CoV-2 infection may be asymptomatic. We will state in our recruitment materials and communications that SARS-CoV-2 symptomatic and asymptomatic participants are eligible, and aim to enroll a sub-cohort of asymptomatic individuals.

5) Importance of including vaccination status in data collection. While this is not specifically a study of vaccination for SARS-CoV-2, the reality is that the upcoming massive vaccination campaign in the United States will serve as the backdrop upon which the study occurs. Because of this, it is likely that at least a fraction of our participants will have been previously vaccinated for COVID, and this proportion is likely to increase over the first 6 months of the study. We will ensure that items regarding COVID-19 vaccination (including timing of vaccination, type of vaccination received, and side effects related to vaccination) are a part of our questionnaires. People who have or have not received a COVID-19 vaccine will be eligible. Given the efficacy of preventing symptomatic COVID-19 reported in Phase III trials of the two currently approved mRNA vaccines, we expect that the numbers of newly recruited COVID+ participants who have already received an mRNA vaccine will be small. However, we expect that a large percentage of COVID+ participants may receive a vaccine in the post-acute phase. This will also allow us to answer important scientific questions that are beyond the scope of the current study but which may be of interest going forward.

Reviewer 3

1. **The research question was designed by amfAR (please see RFP excerpt below). Are the applicants answering the question well?**

I am concerned that the team is reduplicating work that has been done by other groups including the ACTG regarding the effects of COVID19 in persons living with HIV (PLWH). The applicants do not provide sufficient information as to what would be special or specific

for COVID 19 in PLWH compared to having COVID19 in uninfected individuals. The applicants do not control for possible hospitalization or degree of symptoms as this may affect subsequent results. A large list of biological analytes is provided but rationalization as to why they were chosen is not provided. Overall, this seems like more of a fishing expedition. With the large number of tests to be performed there is always the chance that something will be significant. Additional input from statisticians is needed.

2. How useful will the answers be to PWH, or care providers?

I am worried that many of the results will not be that useful to providers. Many if not most of the test administered are questionnaires and are not objective measures of organ function in various participants. For example more objective measure of dyspnea could be obtained instead of subjective measures. The same applies to cognitive evaluations which are not well described nor are sufficient. The applicants state that they will ensure that there is roughly equal groups with regards to race/ethnicity but this seems to be a missed opportunity as there is a large body of work that certain underrepresented minorities are at increased risk for serious consequences from COVID19.

3. Which specific changes should amfAR ask the applicants to make?

I am worried that this study may not be sufficiently powered and that there could be serious limitations in implementation at the sites as discussion as to how to standardize assessment as these important implementation practices are not mentioned. There are numerous missed opportunities to obtain more objective measures that are not obtained. Finally links to existing work that is being performed in the HIV community (e.g. ACTG) should be indentified.

RESPONSE:

We thank Reviewer 3 for their constructive feedback, which we respond to below.

1) Concerns that this work overlaps with ACTG efforts to study PWH and COVID. We are not aware of any other group examining long COVID in PWH at this time. Dr. Landay is on the ACTG leadership team and confirms that the ACTG is not sponsoring any long COVID studies in PWH including in the REPRIEVE or HAILO studies. Given that PWH have elevated levels of T cell activation and higher prevalence of comorbidities despite viral suppression, we hypothesize that PWH are more likely to experience persistent symptoms (long COVID) than HIV-negative people.

2) Concerns about measurement of the effects of long COVID. It is difficult to study this new syndrome in PWH because objective and subjective measures of long COVID are not consistently captured in the electronic medical record, hence large EMR-based studies are not optimal (see response to Reviewer 1, above). Similarly, single-institution studies with objective measures of dysfunction in long COVID (cardiac MRI, pulmonary function tests, 6-minute walk tests, tilt-table testing, etc.) are not feasible for several reasons: 1) even large academic medical centers are likely to capture a limited number of PWH with COVID-19 over a several month

interval, 2) only a subset of such individuals will be willing to enroll in an intensive clinical research study, and 3) PWH served by a single center will likely be limited in diversity and not reflect the true diversity of PWH in the United States. After deliberation among investigators across 6 academic institutions and together with amfAR, we decided that a multi-center approach is the optimal way identify an acceptable number of PWH with COVID coinfection that is more reflective of the geographic and demographic diversity of PWH in the U.S.

While we considered whether we could perform some of the tests cited above on a subset of individuals, the costs of these tests would be prohibitive and would preclude us from the collection of biospecimens unless we reduced the sample size substantially. Therefore, we focus on characterizing the symptoms – in a rigorous way with validated tools that probe the domains of general symptoms such as fatigue – and building a biospecimen repository that the HIV and COVID-19 research communities may access to investigate long COVID in PWH. In addition, we will be able to refer individuals based on their geographic area to local research studies if they are interested in ongoing participation in research.

3) Need for biostatistical support throughout the study. We agree that input from biostatisticians is important. Dr. Antar has an active collaboration to study long COVID in the general population at JHU with two faculty biostatisticians from the Johns Hopkins Bloomberg School of Public Health who are experts in the analysis of survival, longitudinal, and multivariate data (Dr. Mei-Cheng Wang and Dr. Chen Hu) and will seek their support in the analysis of the data from the proposed study. In addition, Dr. Peluso has been working closely with Dr. Jeffrey Martin from the Department of Epidemiology and Biostatistics at UCSF; Dr. Martin co-founded the SCOPE cohort with Dr. Deeks and is a co-investigator with Dr. Peluso on two San Francisco-based COVID-19 natural history studies; he contributed to the sample size calculations in the initial proposal and will provide ongoing support.

4) Selection of specific laboratory tests in the proposal. We selected the specific laboratory tests outlined in our proposal based on a detailed review of the literature in acute COVID-19 and myalgic encephalitis/chronic fatigue syndrome (ME/CFS), which many have noted may be similar or related to long COVID. We were discouraged from including a detailed literature review in the application form but will summarize it here.

First, we reviewed the literature on biomarkers that can discriminate ME/CFS from healthy controls. Toshimori Kitami and colleagues conducted deep phenotyping of ME/CFS vs healthy controls and found that monocyte number, microbiome profiles, and lipoprotein profiles provided the best discriminatory function to identify ME/CFS patients vs controls, hence our inclusion of CBC with diff and lipid panel (Kitami et al. Sci. Rep. 2020). Almenar-Perez and colleagues found that creatine kinase, extracellular vesicles, and a limited number of miRNAs associate with severe ME/CFS, hence our inclusion of CK and PBMC collection (Almenar-Perez et al. Sci. Rep. 2020). CD4+ T cell counts are important in any study of HIV co-infection and low CD4 counts in PWH are associated with poor outcomes in acute COVID-19 (Dandachi et al. CID. 2020). Absolute lymphocyte count, C-reactive protein, and d-dimer are all associated with severity of acute COVID-19 as demonstrated by multiple groups, and the PI has written a review about these lab tests and their associations that is currently under review. IL-6 and TNF-alpha are strong and independent predictors of acute COVID-19 severity and death, even after adjusting for disease severity, other common lab inflammation markers, hypoxia, demographics, and a range of comorbidities (Del Valle et al. Nat. Med. 2020).

Certain cytokine profiles in acute SARS-CoV-2 infection are associated with different disease phenotypes (Lucas et al Nature 2020, Liang et al Nature Medicine 2020, Del Valle et al Nature Medicine 2020). Levels of various interleukins (IL-1a, IL-1b, IL-6, IL-18) and TNF-alpha correlate with disease severity and survival. Furthermore, perturbations in type 1 interferon pathways

appear to be centrally involved in the trajectory of disease. Previous work identified four immune signatures that correlated with distinct trajectories during acute infection: (1) growth factors, (2) type 2/3 cytokines, (3) mixed type 1/2/3 cytokines, and (4) chemokines. It is possible that similar signatures could be identified that are unique to PWH or unique in long COVID.

5) Concern about standardization of assessments. We agree that this is an important point, and want to emphasize that standardization of assessments is built into the study structure. External sites serve as recruitment hubs, but questionnaires and cognitive tests are administered by the same study coordinator no matter the site of recruitment. Blood draws will be contracted to a single mobile phlebotomy company that has a national footprint. Blood will be shipped to the same processing and storage facility, overseen by Dr. Landay at Rush.

Our study instruments are for the most part validated tools that have been rigorously developed and assessed for other disease conditions; no such tools exist yet for COVID, but the investigators have been working for nearly a year to develop and optimize tools in their local COVID studies. In addition, the World Health Organization will soon be releasing recommendations to measure outcomes in long COVID and these will be incorporated when available. In the application, we have listed our validated tools to rigorously assess symptoms and their impact on daily function in the original application. The Fatigue Severity Scale assesses fatigue's effects on daily functioning and queries its relationship to motivation, physical activity, work, family, and social life, and asks respondents to rate the ease with which they are fatigued and the degree to which the symptom poses a problem for them. The Insomnia Severity Index© assess the perceived severity of difficulties initiating sleep, staying asleep, and early morning awakenings. Additionally, it probes satisfaction with current sleep pattern, interference with daily functioning, noticeability of impairment attributed to the sleep problem, and degree of distress or concern caused by the sleep problem.

We agree that assessing the neurologic complications in patients with COVID-19 is critical as numerous studies report anywhere from mild (e.g, headaches, loss of smell/taste) to severe (i.e., stroke, hemorrhagic encephalitis, delirium) neurologic symptoms. In order to successfully integrate neurologic assessments (cognition and mental health) into our proposed project, we have engaged Drs. Leah Rubin, an Associate Professor in Neurology, Psychiatry and Behavioral Sciences, and Epidemiology at JHU and Dr. Tracy Vannorsdall, an Assistant Professor of Psychiatry and Behavioral Sciences and Neurology at JHU. Since the beginning of the pandemic, Drs. Rubin and Vannorsdall have successfully transitioned a number of their studies that required inpatient neuropsychological test battery assessments into phone or tablet-based neurological and mental health assessments. We plan to capitalize on their experience and integrate the following assessments into our study.

1. **MESA telephone neuropsychological test battery** which is geared off of portions of the UDS3 telephone testing battery. It incorporates a measure of learning and memory (Rey Auditory Verbal Learning Test-RAVLT], attention/working memory (digit span task-forward & backward), phonemic and semantic fluency (letter-guided and category-guided fluency tests), and a measure of attention/executive function (oral Trail Making Test Part A and B). This battery is already being used in a number of JHU COVID studies.
2. **Computer Adaptive Test-Mental Health (CAT-MH)**, a highly sensitive and specific internet-based screening tool for a DSM-V diagnosis of major psychiatric disorders. The tool draws upon items from depression, anxiety, post-traumatic stress, and substance use disorder and customizes itself in real- time based upon the participant's answers.

The participant responds to several items in order to arrive at a diagnosis of these disorders. Result categories for substance use disorder include severity and frequency of use for distinct categories of substances: alcohol, sedatives, heroin, methadone, fentanyl, cocaine, amphetamines. We will be able to simply send participants the CAT-MH link for each proposed study visit. This tool has been integrated in a substudy in our NIMH-funded P30 Clinical Core which is assessing COVID, cognitive, and mental health symptoms in our cohort of people with and without HIV.

3. **PHQ-9,** a 9-item self-reported measure of depressive symptoms and the GAD-7, a 7-item self-reported measure of generalized anxiety will also be incorporated into the REDCAP assessment.

Dr. Rubin is also currently working with Joan Severson, founder of Digital Artefacts, to assist in the development of a phone-based (android & iphone) app to assess neurologic complications of COVID (https://www.digitalartefacts.com/). The app will include both neuropsychological tests (e.g., Stroop Test) and a neurological daily symptom diary (e.g., headaches, loss of sense of smell/taste, etc). Unfortunately, the currently available app is only available for use on the iPhone and we recognize that many individuals use Android-based phones. The tool is likely to be ready for use in March 2021. As soon as it is ready, we will integrate it into our proposed study.

Finally, we are enrolling PWH who do not have COVID-19 simultaneously to capture the stress/isolation/impact of living in 2020-2021 even without acquiring COVID-19.

6) Concerns about diversity of the study population. As discussed in our response to Reviewer 2, we will ensure that the study population reflects the age, sex, and race/ethnicity makeup of the population of people living with HIV in the United States.

Reviewer 4

1. **The research question was designed by amfAR (please see RFP excerpt below). Are the applicants answering the question well?**

The research question designed by am far is a request to “document the existence of, and any differences in, the experience of post-viral syndrome in PWH” in comparison to the syndrome in people who are HIV negative.

The applicants are addressing this research question through a comparative exploration of post-acute Covid 19 (PAC 19), including an array of biological markers, amongst people living with HIV as compared to people with PAC19 who are HIV negative. They will also compare with people living with HIV who have no history of Covid 19. I note the absence of a further group of people who are HIV negative and who do not have a history of Covid 19. Such a group would provide a useful baseline and is notable by its absence.

This seems to be an opportunity to explore both the ways in which PAC 19 manifests in people living with HIV and conversely the ways in which HIV might impact on the experience of PAC 19. It is also an opportunity to develop a particularly important cohort of people. The natural history of this condition is still to be determined and future follow-up, beyond 180 days, could be important.

It would be helpful to have more detail on the proposed sampling frame. Although the investigators discuss recruitment hubs that will represent demographic and geographic

diversity and mention of matching later in the protocol it would be useful to have a more detailed breakdown of this aspect of the study. The numbers of participants seem to be on the low side given the diversity of people living with HIV and the diversity of people who experience PAC 19 and the need to control appropriately for a wide range of variables. I note the statistical analyses, however it is important to ensure they are powered to consider potentially relevant demographic and other variables, in particular the presence or absence of comorbidities.

I note that people with HIV who have not had Covid will not be followed up beyond the initial visit. I'm not quite sure why the investigators wish to lose this control group and I would like to understand the justification for this decision.

It would be useful to know more about the treatment and virological outcomes of the people living with HIV who are selected to take part. What will happen if people need to change ARVs during the study?

The design as a prospective study is appropriate.

The team is highly experienced and has all the appropriate skills and experience to undertake the work to time and target.

2. How useful will the answers be to PWH, or care providers?

The answers will be very useful to both people living with HIV, care providers and commissioners of care. Given the scale of the Covid 19 pandemic and the numbers of people who are experiencing PAC 19 this is likely to become very important question which will have specific implications for the well-being of people living with HIV. The development of a study by a repository adds value to this study and could produce useful information.

3. Which specific changes should amfAR ask the applicants to make?

I would like to see additional information about the composition of the study groups as I have set out. This will also need to feed into the statistical analyses. This might in turn change the number and composition of the study groups

RESPONSE:

We thank Reviewer 4 for these suggestions, which we respond to below.

1) Suggestion of the benefit of including HIV-negative, COVID-19 negative comparators. Thank you for this suggestion, which we will incorporate. Our comparison group for those with long COVID or PAC-19 is, ultimately, those people who experience COVID-19 but do not have long COVID or PAC-19, which explains our focus on groups 1 and 2. We think that comparison will be most helpful to determine the mechanistic cause of long COVID. However, Reviewer 4's suggestion is an excellent one. We will recruit a similar number of HIV-COVID- people as HIV+COVID- people (~50) for symptom surveys and cognitive testing. Biological sampling is the most expensive component of our budget, and 50 more biological specimens would not fit within the budget, unless 50 participants are taken from the other groups. However, we will have the ability to leverage cohorts such as MACS/WIHS and SCOPE that contain biospecimens from

HIV negative participants collected prior to 2019 for biological comparisons. This will allow us to easily match demographics with other groups.

2) Necessity of follow-up beyond 4 months. We agree that ongoing follow-up beyond 4 months is important and necessary. The nature of the funding is that no-cost extensions will not be allowed and so we designed a study that can be completed in a year, hence the 4 month time-point, which is also being used as an outcome time point in other studies of long Covid. When we consent participants, we will include a statement saying that they consent to be contacted for future studies. amfAR and other funders may decide to fund additional proposals seeking to follow up this cohort.

3) Concerns about sample size. Our ideal study would include a larger sample, but we are limited primarily by funding and the biospecimen collection arm. Collecting and processing biospecimens is the single largest piece of the proposed budget. While we considered other designs that would allow for a larger study sample, we ultimately felt that biospecimen collection was critical for mechanistic work in PAC-19/long COVID, and we will make it available with a simple request and approval process similar to UCSF's SCOPE cohort.

4) Lack of longitudinal data collection on HIV+/COVID- comparators. Items about HIV care, including ARV change and the reason for it, will be included in the surveys. The reviewer brings up an important point – why group 3 (HIV+COVID-) will not be followed up. Biospecimens will not be collected twice in this group because of the expense and because they will serve as a comparator for the other groups. However, as Reviewer 4 suggests, we will ensure that this group has symptom surveys and cognition testing at 4 months to serve as an important time-varying control.
